# The disease and economic burden of notified and underestimated *Campylobacter* enteritis cases and associated sequelae in Germany

**DOI:** 10.1371/journal.pone.0331298

**Published:** 2025-09-04

**Authors:** Elisabeth Schorling, Sonja Lick, Bettina Rosner, Sebastian Knorr, Hendrik Wilking, Pablo Steinberg, Dagmar Adeline Brüggemann

**Affiliations:** 1 Department of Safety and Quality of Meat, Max Rubner-Institute, Federal Research Institute of Nutrition and Food, Kulmbach, Bavaria, Germany; 2 Department of Infectious Disease Epidemiology, Robert Koch-Institute, Berlin, Germany; 3 Max Rubner-Institute, Federal Research Institute of Nutrition and Food, Karlsruhe, Baden-Württemberg, Germany; National Center for Chronic and Noncommunicable Disease Control and Prevention, Chinese Center for Disease Control and Prevention, CHINA

## Abstract

**Background:**

According to surveillance data, *Campylobacter* enteritis (CE) has been the most frequently notified bacterial gastrointestinal disease in Germany and Europe for many years. Presumably, the total number of cases is underestimated because an unknown number of cases is not diagnosed and some diagnosed cases are not reported in the surveillance system. The aim of this study was to estimate the disease and economic burden of CE and its related sequelae in Germany.

**Methods:**

The disability-adjusted life years (DALY) as well as the direct and indirect costs associated with the five-year (2018–2022) mean number of CE cases and related sequelae were estimated in a Monte Carlo simulation. The age- and gender-specific characteristics were integrated where possible. The underestimated CE cases were quantified by reconstructing the surveillance pyramid using age group-specific health care seeking parameters.

**Results:**

The estimated incidence rate was 553 CE cases (95%-CI: 551–555 cases) per 100,000 inhabitants per year. This corresponds to 7.7 underestimated cases per notified case. Underestimation was lowest in the age group <5 years and highest in the age group 15–29 years. The notified plus underestimated CE cases and associated sequelae resulted in a loss of 6,764 DALY (95%-CI: 6,689-6,839 DALY), 88% of which were due to sequelae. The total economic burden amounted to 263.5 million Euros (95%-CI: 262.5–264.4 million Euros). Approximately 25% of the total DALY and costs were attributable to the notified cases.

**Conclusions:**

The results suggest a substantial burden due to CE, both in terms of DALY and costs, in Germany. Especially the high number of underestimated cases and associated sequelae contribute to the health and economic burden – although some remaining uncertainties cannot be ruled out. By using age-specific multipliers to determine the underestimated cases, age-related differences in DALY and cost of illness can be accounted for, thereby preventing an overestimation of the total burden.

## Introduction

*Campylobacter* enteritis (CE) is the most frequently reported bacterial gastrointestinal infection in humans within the European Union (EU) [1]. In Germany, CE has been notifiable according to the Protection against Infection Act since 2001. The incidence of notified CE cases in Germany peaked in 2016 with 90 cases per 100,000 inhabitants and fell since then to 48 cases per 100,000 inhabitants in 2023 [2,3]. Due to the impact of the COVID-19 pandemic, CE incidence rates decreased by around 25% in 2020 in Germany as well as throughout the EU when compared to the previous year [4,5] and continued to remain at approximately the same level since then [2,6].

*C. jejuni* and *C. coli* are the predominant species causing CE in humans, being mainly transmitted through contaminated broiler meat, but also through contaminated water and raw milk [1,7,8]. Human infections with *Campylobacter* spp. can be associated with (bloody) diarrhea, abdominal pain, fever, nausea, and/or vomiting and may require hospitalization in severe cases. The infections are usually self-limited and the symptoms disappear within two weeks [7,9]. CE cases occur in all age groups, the highest incidence being observed among children younger than five years of age and young adults aged between 20 and 29 years [2,3]. Hospitalizations due to CE are more common among children younger than one year of age and patients older than 70 years of age [10,11]. The case fatality is low with up to ten reported deaths per year in Germany; deaths due to CE usually occur among people being older than 55 years of age [12]. Reported post-infection complications of CE are reactive arthritis (REA), Guillain-Barré syndrome (GBS), inflammatory bowel diseases (IBD) and irritable bowel syndrome (IBS) [9,13,14].

Modern *Campylobacter* epidemiology increasingly relies on whole genome sequencing (WGS) for outbreak tracking and resistance profiling. Emerging evidence from genomic surveillance also highlights the increasing prevalence of antimicrobial-resistant (AMR) *Campylobacter* strains in Europe, thereby complicating the treatment of CE and resulting in a prolonged disease duration [15].

The disease burden caused by *Campylobacter* spp. is estimated to be high in Europe and worldwide [16,17]. Estimates for Germany revealed 8,811 disability-adjusted life years (DALY) due to notified and underestimated CE and sequelae in 2014 [18]. The major part of the disease burden was due to morbidity; premature mortality due to CE itself is rare and tends to occur in elderly patients [18,19].

Our group recently analyzed the cost of illness associated with diagnosed CE and sequelae using claims data of almost 10,000 patients [20]. Based on the notified number of CE cases in Germany in 2017, total costs of up to 95 million Euros were calculated. This amount does not account for the CE cases that were not identified by the surveillance system.

The human infections caused by *Campylobacter* spp., which are not recorded in the surveillance system, may considerably contribute to an underestimation of the real number of infections within a population [21,22]. Underestimated cases comprise i) underascertained cases at the community-level, referring to patients that did not seek medical care, and ii) underreported cases at the medical care-level, referring to patients that did seek medical care but were either not diagnosed or not reported as *Campylobacter*-positive cases. While underascertainment in Germany is mainly attributable to mild symptoms and the self-limiting nature of CE, the underreporting results from a lack of laboratory confirmation. The confirmation (and the subsequent notification) of CE cases depends in particular on the decision of the medical staff to ask for a stool sample examination and the limitations of the current detection methods in the laboratory. The predominant factors influencing the notification of CE cases are summarized in Table 1.

**Table 1:** Factors influencing the notification of CE cases in Germany.

Level	Influencing factors
Local and state health department	• Timely and correct reporting from the local to the state health department and the federal public health institute
Primary diagnostic laboratory	• Timeliness and correctness of stool sample testing: influenced by, e.g., capacity, expertise/ accreditation • Limitations of detection methods (sensitivity, accuracy) • Timely and correct reporting to the local health department
Healthcare institutions (GPs, hospital)	• Initiating a stool sample examination: influenced by, e.g., medical necessity according to national medical treatment guidelines, budgeting • Patient information
Patients	• Presence, duration and severity of symptoms • Health literacy: assessment of duration, severity, self-limitation of symptoms and need for medical care • Access to the health care system • Confidence in the health care system • Correct collection and timely sharing of a stool sample

Factors described by Gibbons et al. and FERG [21,22] were reviewed and adapted to the German context.

The actual number of CE cases in Germany is estimated to be four to nine times higher than the CE cases that are notified within the surveillance system [23,24]. However, these multipliers do not reflect age-specific differences, for example regarding the probability that patients seek medical care if they suffer from gastrointestinal symptoms.

This study aimed to estimate the disease burden and the economic consequences of both notified and underestimated CE cases and associated sequelae in Germany from a societal perspective, using secondary data and published evidence. By applying age group-specific multipliers, we provide refined estimates of underreporting and underascertainment of CE across different age groups in Germany.

## Methods

### Outcome tree of *Campylobacter* enteritis

**Fig 1:**
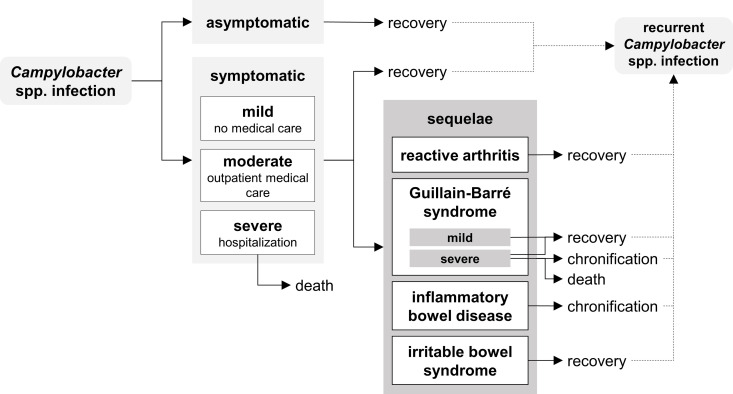
Outcome tree of *Campylobacter* enteritis. Disease outcome tree in line with earlier disease models [18,20,25].

The course of CE and potentially associated sequelae is summarized in a disease outcome tree (Fig 1). In line with earlier disease models [18,20,25], symptomatic CE was categorized into three severity types, depending on the utilization of health care resources as i) mild (no medical care), ii) moderate (outpatient medical care only), or iii) severe (hospitalization, with or without outpatient medical care). Asymptomatic infections are not associated with health or economic consequences and were therefore not considered.

REA and GBS are widely recognized as sequelae of CE. GBS was further divided into the subcategories mild and severe. Severe GBS cases can be temporary, chronic or fatal. Additionally, IBD, i.e. Crohn’s disease (CD) and ulcerative colitis (UC), and IBS were incorporated into the model, although the exact mechanisms by which *Campylobacter* spp. may be involved in the pathogenesis of IBD and IBS are not fully understood up to now [26–29]. It was assumed that deaths due to CE or GBS only occur after severe disease courses.

### Modelling approach

Due to the large variation in the number of CE cases in recent years, the average number of notified cases per year in Germany, calculated for the time period 2018–2022, was used for the estimation of the disease and economic burden (n = 53,578). CE cases in Germany are counted in the national surveillance system if they show the clinical picture of an acute CE, either with an epidemiological (<0.5% of the cases) or a laboratory (>99% of the cases) confirmation of CE [2]. Therefore, all notified cases were categorized as moderate or severe cases.

The extrapolation followed an incidence approach, in which both the current and future health and economic consequences associated with CE cases and subsequent sequelae were assigned to the baseline year 2022 [21]. This year was selected because it was the most recent year in which all relevant official statistical data were available at the time that the analyses were conducted. The course of disease was modelled for average patient groups, defined by age and gender. The age groups (0–4, 5–14, 15–29, 30–44, 45–64, 65–74 and ≥75 years) were selected taking into account i) the high CE incidence rates among small children and young adults [2,3,7], ii) differences in the remaining life expectancy, and iii) age-related differences in the cost of illness [20]. The DALY lost and health care costs incurred in each patient group were analyzed in R 4.4.1. A Monte Carlo simulation with 10,000 iterations was performed using the R package mc2d [30], whereby parameters varied simultaneously depending on a priori defined distributions. All input parameters including the assumed distribution functions are given in the S1 Appendix. The R script is available via OpenAgrar [31].

The age group- and gender-specific numbers of notified CE cases in the German notification system database for the time period 2018–2022 were accessed via SurvStat [2]. Hospitalizations and deaths due to CE in Germany from 2018 to 2022 were taken from official statistics [10–12,32]. The number of moderate cases was calculated by subtracting the number of hospitalized cases from the total number of notified CE cases. Additionally, the underestimated CE cases in Germany were quantified as described below.

The number of sequelae for both notified and underestimated CE cases was assessed by using the pooled estimates of recent meta-analyses [33] as most likely values, and the prediction intervals as the minimum and maximum in a modified PERT distribution. REA, IBD and IBS are more likely to occur as sequelae of CE if CE was prolonged and/or associated with more severe symptoms [20,34–36]. Therefore, it was assumed that only moderate and severe CE cases, but not mild cases, might develop these sequelae.

For GBS, age group- and gender-specific input parameters were integrated into the model. It was assumed that CE-associated GBS cases show the same distribution across age groups and gender as all GBS cases in Germany. The estimated total number of GBS cases in Germany in 2022 (2,300 cases) was based on recent national incidence data [37].

The assumptions regarding the occurrence and severity of sequelae as well as the duration of both CE and sequelae are explained in detail in the S1 Appendix.

### Underestimated *Campylobacter* enteritis cases

The number of underascertained (i.e., mild) and underreported (i.e., moderate or severe) CE cases in Germany were estimated by following the recognized approach for reconstructing the surveillance pyramid by Haagsma et al. [24]. The proposed country-specific and general parameters were reviewed and updated (as explained in the S1 Appendix). Additionally, the model was extended by integrating age group-specific probabilities of seeking health care and submitting a stool sample if symptoms of an acute gastrointestinal illness were present. For the age groups ≥15 years, results of the German Health Update (GEDA) study were used [38], as acute gastrointestinal illness (AGI) was a major topic in the 2009 survey [39,40]. As comparable national data on AGI in children are not available, published probabilities obtained from i) an Italian survey with a similar study protocol as the GEDA study [41] and ii) three hospitals in Germany [42] were taken into account. The parameters and formulas used for the correction of the underestimated CE cases are described in the S1 Appendix.

### Disease burden

The disease burden was evaluated by estimating the disability-adjusted life years (DALY) due to CE and associated sequelae. DALY is a commonly used metric composed of the sum of the years lived with disability (YLD) resembling the morbidity, and the number of years of life lost (YLL) due to premature mortality [21]. For the estimation of morbidity, the time lived with CE or sequelae was valued with a disease-specific disability weight ranging between 0 (no impact on health) and 1 (death), and the number of cases affected. As no specific disability weights have been assessed for the German population up to the present time, published weights based on surveys in Hungary, Italy, the Netherlands and Sweden [43] were used as the most likely, minimum and maximum values in a PERT distribution, as recently proposed by the European Centre for Disease Prevention and Control [25] (see Table F in the S1 Appendix). For the YLL estimation, premature deaths of cases with severe CE or severe GBS were multiplied by the remaining life expectancy in Germany [44]. It was assumed that CE-associated deaths occur at the end of a severe CE. For GBS, the median time from onset of symptoms to death was 33 days, according to the International GBS Outcome Study [45].

### Economic burden

The cost of illness was calculated from a societal perspective, considering direct and indirect costs of the notified and the underestimated CE cases. Direct medical costs for the treatment of CE and sequelae as well as indirect costs due to productivity losses of working patients were derived from a recent analysis of claims data of 9,945 insurants with diagnoses of CE [20]. As some insurants showed multiple CE infections per year, the cost estimates were divided by a uniformly distributed correction parameter, as explained in the S1 Appendix.

For sequelae, the direct costs were limited to medical care in hospital, as these services were directly linked to diagnoses. The estimation of costs for outpatient medical care and prescribed medications (as done for CE) was not possible due to the low number of cases that developed sequelae [20].

The original cost estimates date back to 2017 and were inflation-adjusted to the year 2022 using the harmonized index of consumer prices for Germany [46]. Costs were modelled as PERT distributed with the mean, lower and upper level of the 95% confidence intervals of cost estimates as the most likely, minimum and maximum value (Table G in the S1 Appendix).

Additionally, the productivity losses of mild CE cases and of parents or legal guardians providing informal care of minors suffering from CE or related sequelae were included. These indirect costs were calculated by multiplying the presumed duration and probability of work absence by the average per day labor costs for Germany in 2022, as explained in detail in the S1 Appendix.

For each CE case and CE-related sequela, the corresponding age group- and gender-specific costs per year were multiplied by the disease duration (in years) or, in the case of long-lasting or chronic diseases, by the remaining life expectancy.

### Sensitivity analyses

In order to identify parameters with the highest impact on the final estimates of i) underestimated cases, ii) total DALY and iii) total costs, sensitivity analyses were performed: Correlations between the varying input parameters and the outcomes were calculated and visualized in tornado diagrams. Spearman’s rank correlation was used because it is a non-parametric method. It allows the analysis of relationships between variables without assuming any specific distribution, making it suitable for the diverse sets of input parameters. Due to the remaining uncertainty regarding the development of sequelae, we report results of the disease and economic burden i) without considering IBD and IBS as causal consequences of CE, and ii) under the assumption that REA, IBD and IBS might also develop following mild CE cases. Additionally, DALY and costs in future years were discounted with different rates: In the base case scenario, a rate of 3% was applied, while the sensitivity analyses were performed with rates of 0 and 5% [47].

## Results

### *Campylobacter* enteritis and sequelae cases

According to national surveillance data for the time period 2018–2022, 53,578 CE cases were notified per year on average (arithmetic mean) [2], and 11,114 annual hospitalizations were associated with a principal diagnosis of CE [10,11]. Hospitalized cases were categorized as severe cases, while the remaining 42,464 cases were considered as moderate in this study. On average, six patients per year died due to CE between 2018 and 2022 [12,32].

**Fig 2:**
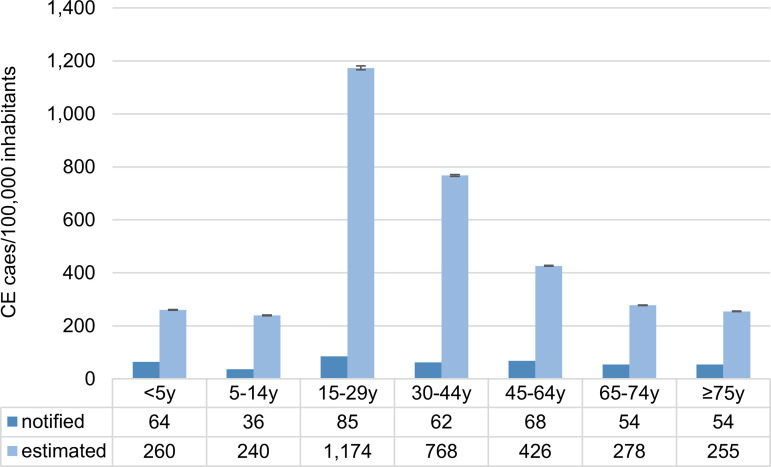
Incidence rate of *Campylobacter* enteritis in Germany by age group based on notifications and estimations. Notified: Incidence rates of *Campylobacter* enteritis (CE) according to national surveillance data, mean annual incidence for the time period 2018-2022 [2]. Estimated: Estimated incidence rates include a correction for underestimated cases.

In addition, underestimated cases were assessed by reconstructing the surveillance pyramid using age group-specific health seeking parameters: 264,561 underascertained (mild) and 148,261 underreported (moderate and severe) CE cases were estimated, resulting in a total of 466,400 CE cases (95% CI: 464,651–468,149 CE cases; Table 2 and Table A in the S2 Appendix). This corresponds to an overall multiplier of 8.7, when comparing the number of notified cases with the total number of estimated cases. Underestimation was lowest in the age group <5 years and highest in the age group 15–29 years (average of 3.0 and 12.8 underestimated cases per notified case, respectively, Fig 2). The resulting mean incidence rate was 553 CE cases (95% CI: 551–555 CE cases) per 100,000 inhabitants, whereas the mean incidence rate of notified CE cases was 64 per 100,000 inhabitants in the time period 2018–2022 [2].

**Table 2:** Disease and economic burden associated with *Campylobacter* enteritis cases.

	notified/ following notified CE	underestimated/ following underestimated CE	total	% of total burden
***Campylobacter* enteritis (CE)**				
**Cases**	53,578	412,822 (411,073-414,571)	466,400 (464,651−468,149)	
**DALY**	199 (199−199)	612 (608-617)	811 (807-816)	12%
YLD	156 (155-156)	584 (580-589)	740 (736-744)	
YLL	43 (43−43)	28 (28−28)	71 (71−71)	
**Total costs** [in € 1,000]	64,847 (64,824−64,871)	151,504 (150,892−152,117)	216,351 (215,736−216,967)	82%
Direct costs	39,564 (39,557−39,572)	61,369 (61,056-61,682)	100,933 (100,620−101,247)	
Indirect costs	25,283 (25,264−25,302)	90,135 (89,769−90,500)	115,418 (115,049-115,787)	
**Sequelae**				
**DALY**	1,502 (1,483−1,521)	4,451 (4,394−4,507)	5,953 (5,878−6,027)	88%
YLD	1,496 (1,477−1,514)	4,369 (4,313−4,425)	5,865 (5,791−5,938)	
YLL	6 (6-7)	82 (80-83)	88 (86-90)	
**Total costs** [in € 1,000]	9,090 (8,990−9,189)	38,012 (37,561−38,463)	47,102 (46,564−47,640)	18%
Direct costs	3,358 (3,319−3,397)	15,239 (15,033−15,445)	18,597 (18,357−18,837)	
Indirect costs	5,732 (5,663−5,800)	22,773 (22,491−23,056)	28,505 (28,161−28,849)	
***Campylobacter* enteritis and sequelae**				
**DALY**	**1,701** **(1,682−1,720)**	**5,063** **(5,006−5,120)**	**6,764** **(6,689−6,839)**	
without IBD/IBS	429 (423-435)	1,508 (1,487−1,528)	1,937 (1,910−1,963)	
undiscounted	3,887 (3,844−3,931)	12,555 (12,405−12,705)	16,442 (16,252−16,633)	
discounted at 5%	1,237 (1,223−1,251)	3,582 (3,540−3,624)	4,819 (4,763−4,874)	
**Total costs** [in € 1,000]	**73,937** **(73,835−74,039)**	**189,516** **(188,654−190,378)**	**263,453** **(262,540−264,366)**	
without IBD/IBS	67,202 (67,150−67,255)	168,600 (167,851−169,348)	235,802 (235,030-236,574)	
undiscounted	85,569 (85,328−85,809)	242,581 (241,179-243,983)	328,150 (326,578−329,721)	
discounted at 5%	71,078 (71,005-71,151)	176,715 (175,954−177,475)	247,793 (247,003-248,583)	

Mean and 95% confidence intervals of 10,000 simulations. Extrapolation is based on the mean of notified *Campylobacter* enteritis (CE) cases between 2018 and 2022. If not stated otherwise, DALY and costs in future years were discounted with 3%.

DALY: disability-adjusted life years; IBD: Inflammatory bowel diseases; IBS: irritable bowel syndrome; YLD: years lived with disability; YLL: years of life lost.

The variability in the proportion of CE cases with bloody diarrhea and in the probability of submitting stool samples in the age group 15–29 years (Spearman’s ρ = −0.79 and −0.77, respectively) had the largest impact on the number of underestimated CE cases, followed by the probability of seeking outpatient medical care or submitting a stool sample in other age groups, and the sensitivity of the laboratory analysis for the detection of *Campylobacter* spp. (Fig A in the S2 Appendix).

IBS was by far the most common sequela with 9,002 estimated cases: 2,387 were notified CE cases that subsequently developed IBS, while 6,615 were considered underestimated CE cases (Table B in the S2 Appendix). For REA and IBD, a total of 3,505 and 1,159 cases, respectively, were estimated. When potential cases of REA, IBD and IBS following mild CE were included, the case number for each sequela increased 2.3-fold. GBS was the rarest sequelae; 35 cases were estimated to have emerged after a notified CE. When including the underestimated CE cases, the number rose to 312 GBS cases in total.

### Disease burden

**Fig 3:**
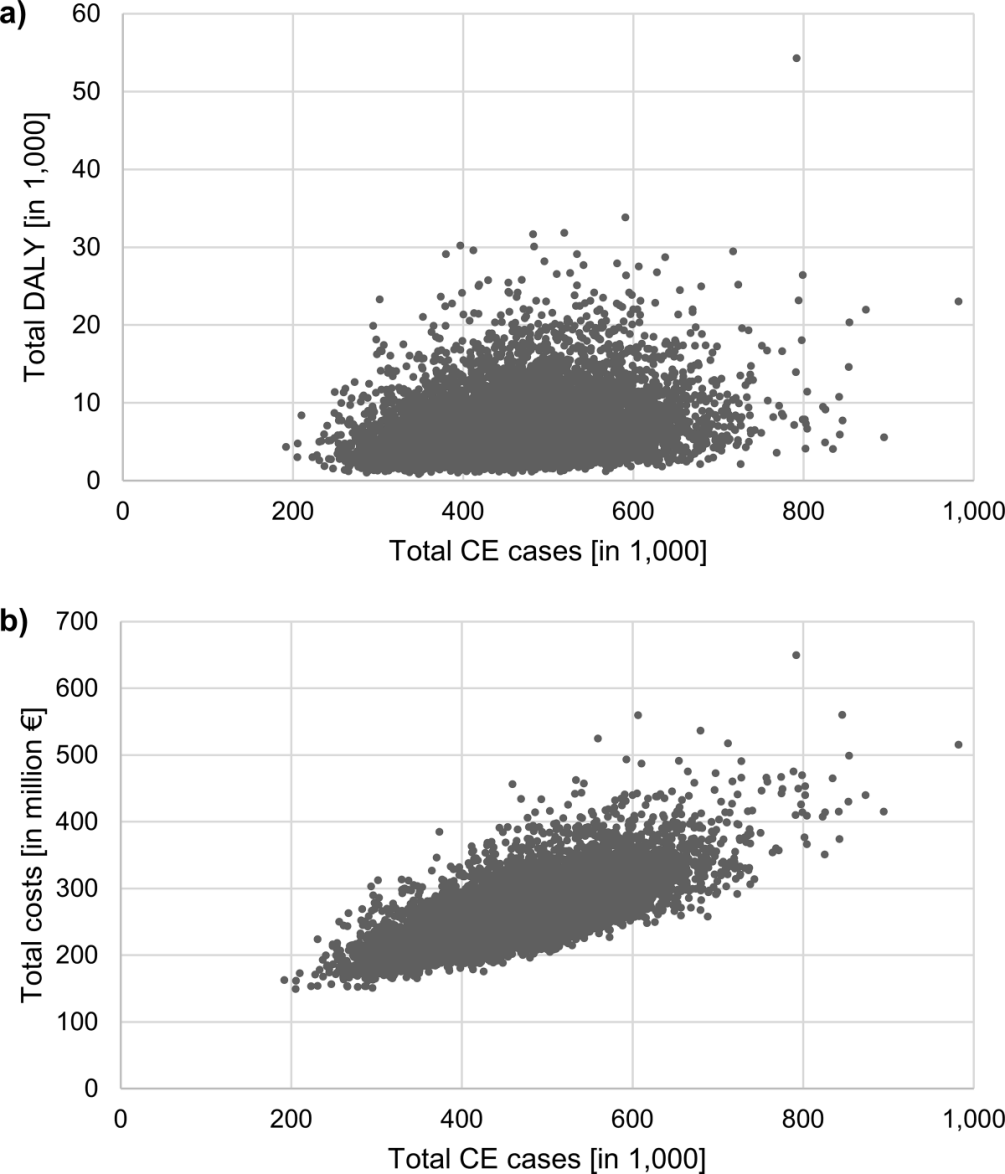
DALY and costs associated with the estimated total number of *Campylobacter* enteritis cases in Germany. Distribution of the total number of *Campylobacter* enteritis cases and A) total DALY, and B) total costs across 10,000 iterations. *Campylobacter* enteritis (CE) cases: sum of notified and underestimated cases; DALY: disability-adjusted life years; costs: sum of direct and indirect costs. DALY and costs in future years were discounted with 3%.

Notified plus underestimated CE cases were associated with a total of 6,764 DALY (95% CI: 6,689−6,839 DALY; Table 2), 88% of them being due to sequelae. The disease burden was mainly driven by morbidity. Premature mortality accounted for 2.4% of the total DALY (71 and 88 YLL due to CE and GBS, respectively). Results of 10,000 iterations of the Monte Carlo simulation are illustrated in Fig 3a.

Of the 811 DALY due to CE alone, about 25% (199 DALY) were caused by CE cases reported within the surveillance system (Table 2). Among notified CE, severe cases accounted for 45% of the DALY (90 out of 199 DALY). When underestimated cases were included, 78% of the DALY were attributable to mild and moderate cases (174 and 462 out of 811 DALY, respectively), and 22% (175 out of 811 DALY) were attributable to severe cases (Table A in the S2 Appendix).

Sequelae after underestimated CE cases increased the disease burden due to sequelae by a factor of four, from 1,502 DALY to a total of 5,953 DALY (Table 2). IBD and IBS were the primary contributors to the overall disease burden (Table B in the S2 Appendix). By excluding both diseases as sequelae of CE, total DALY declined by 71% to 1,937 DALY (95% CI: 1,910−1,963 DALY). In the sensitivity analysis, potential cases of REA, IBD and IBS following mild CE cases were considered and were associated with further 7,375 DALY, resulting in a total of 14,139 DALY (95% CI: 13,962−14,315 DALY) due to CE and sequelae.

The total amount of estimated DALY was strongly influenced by the probability of IBD and IBS, the duration of IBS as well as the multiplier for underestimated CE cases (Spearman’s ρ = 0.26 to 0.45; Fig B in the S2 Appendix). Discounting the disease burden in future years with different rates compared to the 3% rate in the base case scenario resulted in a range of 4,819 (discounted at a 5% rate) to 16,442 (undiscounted) DALY due to CE and sequelae (Table 2).

### Economic burden

The overall economic burden of CE and associated sequelae in Germany amounted to 263.5 million Euros (95% CI: 262.5–264.4 million Euros; Table 2): 82% of the total costs were caused by CE and the remaining 18% by sequelae. Overall, indirect costs accounted for 55% of the total costs, with a slightly higher share among sequelae compared to CE (61% vs. 53%). The estimation of the total costs in 10,000 iterations of the Monte Carlo simulation is shown in Fig 3b.

Similar to the pattern seen for the disease burden, i) the consideration of underestimated CE cases increased the total costs due to CE itself by more than 3-fold when compared to the costs of notified CE cases alone, and ii) severe CE cases accounted for 58% of the economic burden of notified CE cases (37.3 out of 64.8 million Euros). Moderate CE cases were responsible for 53% of the costs when looking at the total number of CE cases (113.7 out of 216.4 million Euros; Table 2 and Table A in the S2 Appendix).

The total costs due to sequelae considerably increased by adding the costs of sequelae of underestimated CE cases, resulting in a total amount of 47.1 million Euros (Table 2). Among sequelae, IBD and GBS contributed the most to the economic burden, each of them with a share of around 7% of the total costs (18.8 and 17.2 million Euros, respectively, Table B in the S2 Appendix). If IBD and IBS were not considered as causal consequences of CE, the sensitivity analysis resulted in slightly lower total costs of 235.8 million Euros (95% CI: 235.0–236.6 million Euros). The consideration of the costs of REA, IBD or IBS cases following mild CE amounted to 40.5 million Euros, resulting in a total economic burden of 304.0 million Euros (95% CI: 302.6–305.3 million Euros) due to CE and sequelae.

The variability of the multiplier for underestimated CE cases (Spearman’s ρ = 0.73) had the largest impact, by far, on the total costs, followed by the probability of GBS, IBD and IBS and the duration of IBS with correlation coefficients of 0.12 to 0.32 (Fig C in the S2 Appendix). The total economic burden discounted at the 0 and 5% rates instead of the 3% rate was 328.1 and 247.8 million Euros, respectively (Table 2).

## Discussion

We estimated the health and economic consequences of CE based on the mean annual number of notified CE cases in Germany between 2018 and 2022. The results show a substantial burden, which was increased in particular by the inclusion of cases that were not captured in the surveillance system (underestimated cases) and of associated sequelae that may develop after *Campylobacter* infections.

### Disease and economic burden comparisons

A previous estimate of the disease burden associated with CE and sequelae in Germany in 2014 by Lackner et al. resulted in 8,811 DALY (undiscounted) based on just over 800,000 estimated CE cases (around 77,200 of which were notified cases) [18]. In the present study, 16,442 DALY (undiscounted) were calculated, including underestimated cases, based on a total of approximately 470,000 CE cases. In contrast to the former approach, in the present study i) we categorized the underascertained and underreported cases as mild, moderate and severe, while Lackner et al. considered all underestimated CE cases to be mild, ii) we assumed that sequelae could also occur in underestimated CE cases, and iii) we integrated new data regarding the disease duration, the disability weights and the probability of developing sequelae. In line with former analyses [18,19], the disease burden due to premature mortality was low and most DALY were lost due to morbidity.

While most DALY were caused by sequelae, the total costs were mainly driven by CE itself. This is also due to the use of partial costs only in the case of sequelae. The true direct costs of sequelae – and therefore the total costs of campylobacteriosis – might be higher.

Our previous estimate of the total costs for almost 70,000 notified CE cases in Germany in 2017 resulted in an amount of 74–95 million Euros [20]. However, this extrapolation i) neglected indirect costs, which incurred due to the caretaking of sick minors and ii) did not account for underestimated CE cases. Furthermore, the evidence regarding the probability to develop sequelae after CE was thoroughly reviewed and new estimates were included [33].

In Germany, coronary heart disease and low back pain caused the greatest number of DALY [48,49]. Communicable diseases accounted for 8% of the total DALY in Germany according to the results of the Global Burden of Disease Study 2021 – mainly due to COVID-19. Diarrheal diseases (including CE) were associated with 72,200 DALY, i.e., 0.24% of the total DALY in Germany [49].

DALY for 30 selected infectious diseases in the EU in 2009–2013 were calculated in the frame of the Burden of Communicable Diseases in Europe study and influenza was the disease with the highest burden, responsible for almost 30% of the total DALY due to the analyzed diseases [17]. Campylobacteriosis had the highest burden among the considered foodborne diseases, accounting for 3% of the total DALY. The calculated 0.01 DALY per CE case (including GBS and REA as sequelae) were slightly lower when compared to the 0.015 DALY per case in the present study. The foodborne disease with the highest burden (3.7 DALY per case) was listeriosis, but with a considerably lower incidence [17]. Taken together, these data show that the burden of CE is mainly driven by the high number of cases. Similarly, the rather modest costs per case for CE and sequelae add up to a substantial economic burden: As seen in other countries, campylobacteriosis belongs to the costliest foodborne diseases – ranking first in Australia and Sweden [50,51] or second after infections due to *Salmonella* spp. in USA [52] and norovirus in Denmark, the Netherlands and the United Kingdom [53–55].

### Underestimation implications

Several factors contribute to the number of underestimated cases, as listed in Table 1. In addition, a considerable decline of notified cases during the COVID-19 pandemic was observed. It remains unclear up to now, how many cases were avoided due to a reduction of risk factors for CE, e.g., eating out or travelling, and how many symptomatic infections were not ascertained due to changes in the health care seeking behavior [56]. In 2020, there was a small decline in the use of outpatient medical care. However, consultations per person had already reached pre-pandemic levels by 2021 [57]. Interestingly, CE notification rates within the EU slightly increased since 2020, but still remain on a considerably lower level compared to pre-pandemic years (61 vs. 46 cases per 100,000 in 2019 vs. 2023) [6]. In Germany, a further incidence decline could be observed since 2021, reaching a new all-time low incidence in 2023 with 48 notified cases per 100,000 inhabitants [2]. Rates increased slightly again in 2024; one of the reasons for this increase is that the case definitions were modified in mid-2023, allowing further laboratory confirmations, e.g., positive PCR testing. This underlines the complex interplay of factors influencing the notification rates and the reconstruction of the surveillance pyramid that persisted beyond the COVID-19 pandemic.

In order to adjust for pandemic- as well as system-related deviations in the notification rates, we based our extrapolation on the average number of notifications between 2018 and 2022, the latter being the most recent year for which the relevant data regarding deaths and hospitalizations were available when conducting the analyses presented in this study.

With a multiplier of 8.7, our analysis of the underestimated cases is similar to the estimate of 9.3 for Germany by Haagsma et al. [24], as they also used the results of the GEDA study for their analysis. However, we were able to integrate for the first time age-specific health care seeking parameters in the extrapolation of underascertainment and underreporting of CE cases. By doing so, major differences in the age group-specific multipliers, ranging from 4.0 referring to patients <5 years of age up to 13.8 in the age group 15–29 years, were observed. CE incidence rates are higher among the youngest according to the national surveillance system [2], which could also be explained by a lower underestimation in this age group.

The correct assignment of underestimated CE cases to age groups is crucial for the analysis of the health and economic consequences, as the remaining life expectancy has an influence on the DALY lost in chronic or fatal diseases, and direct and indirect costs differ depending on age [20,21]. Although the overall multiplier was almost 9, the consideration of underestimated CE cases increased the total DALY and costs by a factor of only 4.0 and 3.6, respectively. This emphasizes the importance of using age-specific multipliers for underestimated cases to prevent an overestimation of the total disease and economic burden.

The most appropriate multiplier should not only be disease-, country- and age-specific, but also gender-specific [22]. Our reconstruction of the surveillance pyramid did not account for gender-specific probabilities, as there were almost no statistical significant differences between men and women with self-reported AGI regarding symptoms and health care seeking behavior in Germany; only the proportion of respondents providing a stool sample was higher in females when compared to men [39]. This is rather surprising, if we consider what we know about gender-related differences in health care utilization: Women usually report more consultations of medical professionals than men [58]. This should be addressed in future studies providing current data on health care seeking behavior. If differences between men and women with AGI emerge, it will be necessary to incorporate gender-specific data into the multipliers.

According to Gibbons et al., reconstructions of the surveillance pyramid lead to higher multipliers compared to other approaches, which can be explained by the more thorough reconstruction of each incremental step of the surveillance pyramid. However, this also means that double-counting of cases cannot be completely ruled out [22]. For example, the estimation based on the disease risks in returning Swedish travelers resulted in only half as many underestimated cases (multiplier of 4.4 for Germany in 2009) [23].

### Methodological limitations

In addition to the issues already mentioned, there are further limitations to our approach: We integrated the available evidence regarding the distribution of input parameters, reflecting either the natural variability or remaining uncertainty. This resulted in wide estimates of the health and economic consequences, as illustrated in Fig 3. Both the total DALY and total costs were most influenced by the variability of the multiplier for underestimated CE cases and the probability and duration of sequelae. The time period we chose in our study (2018−2022) was not optimal because it included the COVID-19 pandemic years, which, as previously explained, affected health care seeking and further behaviour as well as the notification of gastrointestinal diseases in the surveillance system and other factors on which we based our estimations.

The sensitivity analyses showed high variabilities due to the remaining uncertainty regarding the true number of associated sequelae. By including the probability of the development of either REA, IBD or IBS following mild, i.e., underascertained, CE cases, the total DALY more than doubled, while the total costs rose by 15%. In contrast, if IBD and IBS were not considered as causal consequences of CE, less than one third of the total disease burden would remain, while the total costs would be less affected. Although a causal link between *Campylobacter* infections and IBD or IBS is not yet fully established [26–29], epidemiological data strongly suggest an association [59–62]. Based on this evidence, it seems reasonable to consider IBD and IBS as sequelae. However, further research is needed to clarify the relationship between *Campylobacter* and sequelae [33]. This would enhance the reliability of burden estimates.

Around one third of the GBS cases in adults [63] and less than 10% of the GBS cases in children [64] might be associated with previous *Campylobacter* infections. Based on the estimated total number of 2,300 GBS cases in Germany, we would expect around 700 GBS cases following CE. That is more than twice as many as the extrapolated 312 GBS cases that we estimated in this study based on our recent meta-analysis [33]. This confirms that we have chosen a rather conservative approach, which reflects the remaining data gaps related to the occurrence of sequelae.

If available, we considered the newest country-specific data. However, data regarding the health care seeking behavior in individuals with AGI were collected in 2009, data about disease duration of notified CE cases were collected between 2011 and 2014 and the cost of illness estimates were from 2017. While in the case of the cost data annual inflation rates were taken into account to reflect increasing costs, we assumed that the disease duration and the health care seeking behavior were still the same due to a lack of newer data. For children, national data regarding the health care seeking behavior are not available. Therefore, we used results of an Italian survey; whether these data indeed apply to Germany is uncertain at the present time. However, a comparison between the overall consultation rates of medical doctors in Germany and Italy in 2022 were found to be similar, with ten consultations per inhabitant per year on average [65]. The lack of (recent) data is a limitation of our analysis that should be considered when interpreting the results. In order to better understand and account for potential influencing factors in the estimation of multipliers, further observational studies regarding the health care utilization of individuals with AGI and CE patients are necessary.

Moreover, our model does not explicitly reflect the growing relevance of antibiotic resistant *Campylobacter* spp. In 2023, EFSA reported ‘extremely high levels’ of resistance of *C. jejuni* and *C. coli* to ciprofloxacin [15], which was still the most frequently prescribed antibiotic in CE cases in Germany in 2017 [20]. It remains unclear at the present time to what extent increasing AMR in bacteria might have led to prolonged or more severe courses of the disease, or interfered with the treatment of CE since then. The increasing AMR in bacteria is leading to new medical care strategies and requires medical care providers to quickly adapt to this new situation. Therefore, enhanced bacteriological surveillance as well as a regular update of relevant input data (i.e., regarding disease duration, disability weights and health care utilizations) seems necessary, in order to rule out a possible underestimation of the disease burden and associated economic consequences of CE.

Finally, our disease model simplifies the reality, as it does not include a time component. We assumed that all cases of CE and sequelae occurred within one year and modelled the health and economic consequences accordingly. Seasonality of *Campylobacter* infections with higher peaks in summer and the first days of a new year were not taken into account in our models [3,7,66].

## Conclusions

CE and associated sequelae lead to a high disease and economic burden in Germany. Although some uncertainty regarding the true number of underestimated CE cases and sequelae following CE remain, the presented estimates show a considerable potential for health and economic savings if human *Campylobacter* infections could be prevented in Germany. Since *Campylobacter* infections in humans are mainly caused by contaminated food items, in particular contaminated fresh chicken meat [8,67,68], appropriate control measures could contribute to a considerable decrease of acute *Campylobacter* infections and sequelae, and consequently, of disease burden. We estimated the highest incidence rates for the group being 15–44 years of age. This finding indicates that there is a need to implement particular measures for this high-risk group, e.g., increasing knowledge of contamination routes and self-protection as well as ensuring good kitchen hygiene in canteens and the catering industry.

Our results further confirm that appropriate multipliers for the correction of underestimated CE cases should be age-specific. This is particularly true if the resulting case numbers are used to estimate the disease and economic burden: Age-related differences in the health care seeking behavior, the cost of illness and the remaining life expectancy can be accounted for, thereby preventing an overestimation of the total burden.

## Supporting information

S1 Appendix Additional methods. (PDF)

S2 Appendix Additional results. (PDF)
