# Supplementary material for: The disease and economic burden of notified and underestimated Campylobacter enteritis cases and associated sequelae in Germany
Source: PLoS One. 2025 Sep 4;20(9):e0331298. doi: 10.1371/journal.pone.0331298 (PMC12410716; doi:10.1371/journal.pone.0331298)
Supplement: S1 Appendix — (PDF) [file pone.0331298.s001.pdf]

Additional methods

for the disease and economic burden of

Campylobacter enteritis in Germany

by Schorling E, Lick S, Rosner B, Knorr S, Wilking H, Steinberg P, Brüggemann DA

I. Input parameters and distribution functions

1) Population

Table A. Population and life expectancy in Germany in 2022

Age group	Population[1]		Average life expectancy[2] *[in years]*	
	male	female	male	female
0 to 4 years	2,047,036	1,941,645	76.5	81.3
5 to 14 years	4,079,618	3,860,678	69.2	74.1
15 to 29 years	7,010,002	6,468,237	56.4	61.2
30 to 44 years	8,327,474	8,076,304	42.4	46.8
45 to 64 years	11,882,128	12,005,697	25.8	29.7
65 to 74 years	4,429,988	4,970,646	14.8	17.5
≥75 years	3,782,806	5,476,586	7.4	8.3
total	84,358,845			

[1] Population as of 31st of December 2022 [1].
[2] Life expectancy per year of age [2] weighted by the population share in the respective group [1].

2) *Campylobacter* enteritis

Cases

Table B. Mean notified *Campylobacter* enteritis cases, hospitalizations and deaths 2018-2022

	Notified cases		Hospitalizations				Deaths	
			cases		days			
	male	female	male	female	male	female	male	female
0 to 4 years	1,462	1,104	335	243	985	720	0	0
5 to 14 years	1,721	1,170	444	327	1,161	890	0	0
15 to 29 years	5,939	5,484	1,239	1,226	3,936	3,894	0	0
30 to 44 years	5,439	4,774	789	659	2,895	2,387	0	0
45 to 64 years	8,984	7,239	1,378	1,068	6,477	4,984	1	0
65 to 74 years	2,706	2,390	699	574	4,044	3,276	0	0
≥75 years	2,462	2,562	1,050	1,083	7,357	7,760	2	3
other / unknown[1]	142		-	-	-	-	-	-
total	53,578		11,114		50,766		6	

Mean number of notified cases of *Campylobacter* enteritis (CE) [3], hospitalizations due to a principal diagnosis of CE (ICD-10 code A04.5) [4,5] and deaths associated with a CE diagnosis (ICD-10 code A04.5) [6,7] in Germany 2018 to 2022.
[1] Notified cases of unknown age and/or other or unknown gender were classified as moderate CE.

Disease duration of *Campylobacter* enteritis

Mild *Campylobacter* enteritis: The mean duration of acute gastrointestinal illness in cases who did not seek medical care was 3.3 days (median: 2, range: 1-30 days) according to a separate analysis of data of the German Health Update (GEDA) study 2009 [8] for this study. The duration of mild *Campylobacter* enteritis (CE) cases was therefore modelled as exponential distribution with a rate of 1/3.3.

Moderate and severe *Campylobacter* enteritis: The median duration of CE symptoms in notified cases was six days (interquartile range: 5-9 days) according data obtained from CE cases in a case-control study conducted in Germany [9], with minor differences between the age groups (Table C). Differences between non-hospitalized and hospitalized cases were not statistically significant. The duration of moderate and severe cases was modelled as PERT distribution with the minimum, median and maximum number of days according to Table C as the minimum, most likely and maximum value. If the age- and gender-specific duration of hospitalization per case (approximated by dividing the total days in hospital by the hospitalized cases in each group, cf. Table B) was higher than the reported age-specific median duration of symptoms (Table C), the duration of hospitalization was used as the most likely value for symptom duration in severe CE cases.

Fatal *Campylobacter* enteritis: It was assumed that CE-associated deaths occur after severe CE, cf. [10,11].

Table C. Duration of diarrhea in notified *Campylobacter* enteritis cases who participated in a case-control study in Germany, 2011-2014

	Days	
	**median**	**interquartile range**
0 to 14 years	6	4-7
15 to 29 years	7	5-9
30 to 44 years	6	4-9
45 to 64 years	6	4-10
≥65 years	6	4-8
total[1]	6	5-9

Number of days with diarrhea (or other CE-related symptoms if patients did not report diarrhea) in notified non-hospitalized and hospitalized *Campylobacter* enteritis cases according data from a case-control study conducted in Germany, 2011-2014 [9]. Symptom duration was calculated for study cases who were no longer symptomatic at the time of the case interview, and if the time period between symptom onset and case interview was shorter than 60 days (n=1,474).

[1] Used as duration for notified cases of unknown age and/or gender.

3) Sequelae

Estimation of sequelae following *Campylobacter* enteritis

Table D. Probability to develop sequelae after *Campylobacter* enteritis

Sequela	Probability of developing sequela			shape parameter in modified PERT distribution
	most likely	min	max	
Reactive arthritis	1.72 %	0.034 %	47.65 %	27
Guillain-Barré syndrome	0.07 %	0.003 %	1.59 %	23
Crohn's disease	0.22 %	0.002 %	20.69 %	95
Ulcerative colitis	0.35 %	0.003 %	28.16 %	79
Irritable bowel syndrome	4.48 %	0.092 %	70.62 %	15

Probabilities according to meta-analyses (pooled estimate and prediction interval) [12].

For **Guillain-Barré syndrome (GBS)**, age- and gender-specific input parameters were integrated into the model. It was assumed that CE-associated GBS cases show the same distribution across age group and gender as all GBS cases in Germany. The total number of GBS cases in Germany in 2022 was estimated according to recent national incidence estimates [13] applied to the population in Germany [1] (Table E). Age- and gender-specific mortality rates of GBS patients were calculated by dividing the reported deaths due to GBS [6] by the estimated total mean (min, max) GBS cases in Germany. GBS-specific mortality rates were modelled as PERT distribution. 17 % of CE-associated GBS cases were considered as mild [14] (no variation modelled, cf. [10,11]).

Table E. Estimated GBS cases and reported deaths due to GBS in Germany

Age group	Estimated GBS cases *[mean (min-max)]*		Reported deaths due to GBS *[mean (min-max)]*	
	male	female	male	female
0 to 4 years	19 (6-44)	7 (2-26)	0 (0-0)	0 (0-0)
5 to 14 years	31 (16-56)	40 (22-68)	0 (0-0)	0 (0-0)
15 to 29 years	84 (53-128)	84 (55-124)	0 (0-1)	0 (0-0)
30 to 44 years	211 (156-279)	113 (80-156)	1 (0-1)	1 (1-1)
45 to 64 years	505 (409-617)	287 (224-363)	5 (1-8)	4 (2-6)
65 to 74 years	277 (226-338)	197 (156-246)	14 (10-17)	5 (2-6)
≥75 years	208 (142-298)	264 (194-351)	34 (30-39)	29 (23-36)
total	2,327 (1,741-3,094)		93 (69-115)	

Estimated cases of GBS according to recent national incidence estimates [13] (age- and gender-specific rates per personal communication) applied to the population in Germany 2022 [1], and mean reported deaths associated with a diagnosis of GBS (ICD-10 code G61.0) in Germany 2018 to 2022 [6,7].

Disease duration of sequelae

In previous analyses, the duration of **reactive arthritis** (REA) following an infection with *Campylobacter* spp. was modelled as exponential distribution with a mean duration of 222 days [15]. Since then, no new studies reporting the average disease duration of REA following CE were published. Therefore, an exponential distribution with a rate of 1/222 days was assumed.

The duration of **Guillain-Barré syndrome** (GBS) in children was derived from multicenter studies performed in German speaking countries. The median duration from onset of GBS symptoms to the day of no symptoms was 66 days (range: 2-790 days) in children aged one to 16.5 years [16]. In GBS cases with a preceding infection with *Campylobacter* spp. the median duration was 202 days (interquartile range: 121-421 days) [17]. The shorter duration was used for mild GBS cases, modelled as exponential distribution with a rate of 1/66 days. For severe GBS in children, a rate of 1/202 days was considered.

For GBS in adults it was assumed that 17 to 31 % of severe cases remain with permanent disability [11,14] (modelled as uniform distribution). For chronic GBS cases, the remaining age- and gender-specific life expectancy was used as the duration of disease.

The duration of non-chronic GBS in cases ≥15 years was modelled as exponential distribution with a rate of 1/193 days, based on reported proportions of recovered patients by time: It was assumed that a mean of 24 % [11,14] of the patients are chronic cases, 35 % recover within one year [18] and the disease duration of the remaining 41 % was set to a maximum of 52 weeks. According to the International GBS Outcome Study 0 % of the patients reached a F-score of 0 after one week, 1 % after two weeks, 5 % after four weeks, 28 % after 26 weeks, 35 % after 52 weeks [18], resulting in an average disease duration of 27.6 weeks or 193 days[1].

Deaths due to GBS occurred after a median of 33 days (range: 6-280 days) after GBS onset according to the International GBS Outcome Study [18]. The disease duration of severe fatal GBS cases was therefore modelled as exponential distribution with a rate of 1/33 days.

Inflammatory bowel diseases (IBD) are chronic diseases [19,20] and the remaining age- and gender-specific life expectancy was used as the duration of disease.

The symptoms of a post-infectious **irritable bowel syndrome** (PI-IBS) resolve over time [21]. In recent studies of the persistence of PI-IBS over time, approximately 50 % of cases recovered after four to six years [22–24]. The disease duration was therefore modelled as exponential distribution with a rate of 1/5 years; in cases where the modelled disease duration exceeded the remaining life expectancy, the latter was used instead.

[1] Based on an average disease duration of 1.5 weeks in 1 % of GBS cases, three weeks in 4 %, 15 weeks in 23 %, 39 weeks in 7 % [18] and 52 weeks in 41 %.

4) Disability weights

Table F. Disability weights used for the estimation of years lived with disability (YLD)

	Disability weight		Health state
	**mean**	**95 % UI**	
***Campylobacter* enteritis**			
mild	0.073	0.061-0.092	Diarrhea, mild
moderate	0.149	0.120-0.182	Diarrhea, moderate
severe	0.239	0.202-0.285	Diarrhea, severe
**Reactive arthritis**			
	0.344	0.300-0.391	Musculoskeletal problems, generalized, moderate
**Guillain-Barré syndrome**			
mild	0.053	0.042-0.064	Motor impairment, moderate
severe	0.520	0.465-0.581	Spinal cord lesion at neck level (treated)
severe, chronic	0.421	0.377-0.477	Motor impairment, severe
**Inflammatory bowel disease**			
	0.221	0.184-0.260	Crohn's disease or ulcerative colitis
**Irritable bowel syndrome**			
	0.062	0.050-0.077	Irritable bowel syndrome

Disability weights assessed in four European countries (Hungary, Italy, the Netherlands and Sweden) [25]. The reported mean, lower and upper level of the uncertainty interval (UI) were used as most likely, minimum and maximum value in a PERT distribution.

5) Cost of illness

Direct and indirect costs per case according to claims data

Direct medical costs for the treatment of CE and sequela as well as indirect costs due to productivity losses in working patients aged 15 to 64 are based on claims data of 9,945 insurants with CE in 2017 [26]. Costs were inflated to 2022 using the harmonized index of consumer prices for Germany [27] (Table G). Where available, the mean, lower and upper level of the 95 % confidence interval of the cost estimates were modelled as the most likely, minimum and maximum value in a PERT distribution. Otherwise the mean estimate was used.

Insurants with moderate CE had on average 1.11 separate CE diagnoses per capita per year. Severely affected patients had 1.01 separate CE diagnoses per capita per year [26]. Therefore, cost estimates were divided by a correction parameter modelled as uniformly distributed between 1.00 and 1.11 or 1.01, respectively.

Table G. Annual per patient costs of *Campylobacter* enteritis and sequelae in 2022

	Direct costs [in €, mean (95 % CI)]		Indirect costs [in €, mean (95 % CI)]	
	**male**	**female**	**male**	**female**
**moderate CE**				
0 to 4 years	195	250	-	
5 to 14 years	159 (159-160)	190	-	
15 to 29 years	162 (162-163)	194	443 (346-573)	362 (281-476)
30 to 44 years	184	219	634 (481-892)	580 (447-846)
45 to 64 years	218	257	650 (535-813)	577 (483-697)
≥65 years	339	398	-	
total[1]	242 (241-244)		-	
**severe CE**				
0 to 4 years	2,525 (2,427-2,642)	2,481 (2,386-2,577)	-	
5 to 14 years	2,379 (2,285-2,544)	2,416 (2,324-2,645)	-	
15 to 29 years	2,476 (2,335-2,944)	2,360 (2,308-2,429)	670 (560-800)	512 (428-605)
30 to 44 years	2,584 (2,439-3,041)	2,419 (2,341-2,526)	1,312 (1,082-1,593)	967 (753-1,325)
45 to 64 years	2,797 (2,679-2,992)	2,748 (2,646-2,963)	1,286 (1,083-1,550)	1,245 (954-1,795)
≥65 years	3,177 (3,027-3,418)	3,097 (2,989-3,259)	-	
**Reactive arthritis**				
0 to 14 years	870 (284-2,008)		-	
15 to 64 years			235 (0-783)	
≥65 years			-	
**Guillain-Barré syndrome**				
0 to 14 years	12,976 (5,485-24,046)		-	
15 to 64 years			20,158 (0-49,666)	
≥65 years			-	
**Crohn's disease**				
0 to 14 years	292 (140-555)		-	
15 to 64 years			1,402 (706-3,150)	
≥65 years			-	
**Ulcerative colitis**				
0 to 14 years	537 (326-1,225)		-	
15 to 64 years			1,062 (663-1,992)	
≥65 years			-	
**Irritable bowel syndrome**				
0 to 14 years	47 (24-95)		-	
15 to 64 years			263 (165-448)	
≥65 years			-	

Mean (95 % confidence interval, CI) costs according to claims data of 9,945 insurants with *Campylobacter* enteritis (CE) in 2017 [26], inflated to 2022 using the harmonized index of consumer prices for Germany [27].
[1] Used for the direct costs of notified cases of unknown age and/or gender.

Indirect costs of mild *Campylobacter* enteritis cases and of caregivers for sick minors

Indirect costs of moderate and severe cases are based on claims data [26], as described above. Indirect costs of cases with mild CE aged 15 to 64 years and of caregivers for sick minors were calculated based on the assumed duration and frequencies of absence from work per year. Each day of absence from work was charged with € 309 for men and € 248 for women, based on the average labor costs and working time in Germany: In 2022, the labor costs were € 39.80 per hour worked [28] and the weekly working time was 38.8 and 31.1 hours for men and women aged 15 to 64 years, respectively [29]. It was assumed that the working time is distributed over a five-day week.

Mild *Campylobacter* enteritis cases

Absence from work were reported by 14.9 % of respondents with acute gastrointestinal illness aged 18 to 64 years, who did not seek medical care according to the data of the German Health Update (GEDA) study 2009 [8]. The mean duration of absence from work was 2.3 days (median: two, range: one to 14 days) in this group.

In Germany, a medical certificate of incapacity for work is required after three days at the latest according to §5 *Entgeltfortzahlungsgesetz (Continued Remuneration Act)*, which regulates the payment of remuneration in the event of sickness. For mild CE cases aged 15 to 64, it was therefore assumed that work absences occur in 14.9 % of cases with a most likely duration of 2.3 days, modelled as PERT distribution with one and three days as the minimum and maximum value.

Caregivers for sick minors

For sick minors <5 years it was assumed that one parent or legal guardian stays with the child for the duration of the disease; in the case of older children, parents' productivity losses were calculated only for hospitalized cases, for the duration of the hospitalization (Table H). The mean employment rates of parents were used as an approximate value of the frequency of work absences [30]. To prevent an overestimation, the lower employment rates of mothers with young children were used.

As the duration of absence from work of caregivers for children with sequelae is unknown, the age-specific average duration of sequelae-associated hospital stays was calculated by dividing the total days in hospital by the hospitalized cases according to official statistics [4,5]. For the age group 5 to 14 years, the proportion of hospitalizations was approximated by dividing the hospitalized cases due to a principal diagnosis of sequelae (ICD-10 codes M02.1, G61.0, K58, K50.0, K50.1, K50.9 and K51, respectively; mean 2018 to 2022 [4,5]) by the estimated number of sequelae cases in Germany according to age-specific prevalence estimates [31–33] (applied to the total number of children aged 5 to 14 years in Germany in 2022 as listed in Table A). In the case of GBS, current incidence estimates were used to estimate the number of cases [13], in accordance with the approach used in the Global

Burden of Disease Study 2019 [34]. A limitation of this approach is that the statistics are not adjusted for readmissions [4,5], which may lead to an overestimation of the proportions of hospitalization. The resulting input parameters for the age group 5 to 14 years were:

- reactive arthritis: 11 (range: 8-16) hospitalized cases [4,5] among 1,121 (95 % CI 727-1,901) cases (prevalence rate of 14.8/100,000 [33])
- Guillain-Barré syndrome: 73 (range: 56-96) hospitalized cases [4,5] among 71 (95 % CI 38-124) cases (see Table E)
- Crohn's disease: 891 (range: 774-983) hospitalized cases [4,5] among 2,257 (95 % CI 1,873-2,737) cases (prevalence rate of 9.2 to 50.8/100,000 [31])
- ulcerative colitis: 1,259 (range: 1,127-1,369) hospitalized cases [4,5] among 1,356 (95 % CI 1,055-1,753) cases (prevalence rate of 7.8 to 28.2/100,000 [31])
- irritable bowel syndrome: 201 (range: 136-284) hospitalized cases [4,5] among 227,249 (95 % CI 151,499-302,999) cases (prevalence rate of 3.0 % [32])

Table H. Assumed duration and frequencies of absence from work of caregivers for sick children per year

Absence from work	Reference	Distribution
**Caregivers for sick children < 5 years**		
**Mild, moderate, severe CE:** one parent stays with the child for the duration of disease		
**Sequelae:** one parent stays with the child for:		
- REA: 3.0 (1.7-4.1) days<br>- GBS: 11.3 (9.8-13.3) days<br>- CD: 6.2 (3.9-12.5) days<br>- UC: 4.7 (3.9-6.0) days<br>- IBS: 2.6 (2.2-3.4) days	Age-specific duration of disease-associated hospitalization, mean 2018-2022 [4,5]	PERT
in 32.8 to 66.3 % of cases	Employment rate in women with children aged <3 and 3 to 5 years, respectively [30]	Uniform
**Caregivers for sick children 5 to 14 years**		
**Mild/moderate CE:** no absence from work		
**Severe CE/sequelae:** one parent stays with the child in the case of hospitalization:		
- severe CE: 100 % hospitalized (per definition)<br>- REA: 1.0 (0.4-2.2) % hospitalized<br>- GBS: 100 (45.2-100) % hospitalized<br>- CD: 39.5 (28.3-52.5) % hospitalized<br>- UC: 92.8 (64.3-100) % hospitalized<br>- IBS: 0.09 (0.04-0.19) % hospitalized	Estimated sequelae-specific proportion of hospitalized cases (as described above) [1,4,5,13,31–33]	PERT
for the mean duration of the hospitalization:<br>- severe CE: 2.6-2.7 days<br>- REA: 4.6 (2.7-7.1) days<br>- GBS: 11.0 (10.1-12.8) days<br>- CD: 3.7 (3.5-3.9) days<br>- UC: 4.0 (3.8-4.5) days<br>- IBS: 2.4 (2.3-2.6) days	Age-specific duration of disease-associated hospitalization, mean 2018-2022 [4,5]	PERT
in 72.4 to 74.7 % of cases	Employment rate in women with children aged 6 to 9 and 10 to 14 years, respectively [30]	Uniform

CD: Crohn's disease; GBS: Guillain-Barré syndrome; IBS: irritable bowel syndrome; REA: reactive arthritis; UC: ulcerative colitis

II. Reconstruction of the surveillance pyramid

The reconstruction of the surveillance pyramid followed the approach of Haagsma et al. [35] and was extended by integrating age-specific parameters, as displayed in Table I.

1) Age-specific parameters

Adults

For the age groups ≥15 years, results of the German Health Update (GEDA) study were used. In this cross-sectional study telephone interviews were conducted with adults in Germany to gather information on health and disease [36]. In 2009, acute gastrointestinal illness (AGI) was a major topic of the study. Questions asked encompassed, among others, the occurrence and frequency of AGI symptoms as well as associated GP visits and hospitalizations and if stool samples were submitted for microbiological examination [37]. The original study data [8] were re-evaluated for this study to match the defined age groups. Due to small case numbers, study participants ≥65 years were analyzed as one group. The distribution functions were estimated as Beta(s+1, n-s+1).

Children

Comparable country-specific data for children are not available. Therefore, results of a similar survey from Italy [38] were used to obtain the probability of visiting a GP in children with AGI. A comparison between the overall consultation rates of medical doctors in Germany and Italy were found to be similar, with ten consultations per inhabitant per year on average [39]; the same applies to the reported probability of visiting a GP in young adults with AGI (42.6 % of AGI cases in the age group 10-24 years in Italy [38] and 40.6 % of AGI cases aged 18-29 years according to the German GEDA study).

The probability of submitting a stool sample in hospitalized patients with AGI in children <5 years of age was taken from a study analyzing hospital records in three hospitals in Germany [40]. As a similar analysis for older children is lacking, it was assumed that the probability of submitting a stool sample in hospitalized children aged 5-14 years ranges uniformly between the mean probabilities of cases aged 18-29 years (37.5 % according to GEDA study data) and that of cases <5 years (70.9 % [40]).

Similarly, there is no (national or international) data of the probability of submitting a stool sample at GP in children with AGI. Therefore, a uniform distribution between the mean probabilities of cases aged 18-29 years (50.0 % with bloody diarrhea and 14.5 % with non-bloody diarrhea according to GEDA study data) and the mean probabilities of submitting a stool sample in hospitalized children with AGI (70.9 % in the age group <5 years [40] and a mean of 54.2 % in the age group 5-14 years) was assumed.

Table I. Distribution functions of age-specific parameters for the reconstruction of the surveillance pyramid

	Probability of visiting a GP		Probability of submitting a stool sample		
	**with bloody diarrhea** GP_{bd}	**with non-bloody diarrhea** GP_{nbd}	**at GP, with bloody diarrhea** st_{bd}	**at GP, with non-bloody diarrhea** st_{nbd}	**in hospitalized patients** st_{hosp}
0-4 years	Beta(19,8)	Beta(19,8)	Uniform(0.50,0.71)	Uniform(0.15,0.71)	Beta(4881,2005)
5-14 years	Beta(12,11)	Beta(12,11)	Uniform(0.50,0.54)	Uniform(0.15,0.54)	Uniform(0.38,0.71)
15-29 years[1]	Beta(9,6)	Beta(137,207)	Beta(5,5)	Beta(19,107)	Beta(4,6)
30-44 years	Beta(17,9)	Beta(157,320)	Beta(5,9)	Beta(33,109)	Beta(13,5)
45-64 years	Beta(18,9)	Beta(178,277)	Beta(7,7)	Beta(68,93)	Beta(14,3)
≥65 years	Beta(7,3)	Beta(108,117)	Beta(4,3)	Beta(38,55)	Beta(10,3)
unknown[2]	Beta(48,24)	Beta(577,918)	Beta(18,21)	Beta(155,361)	Beta(38,14)
Reference	*[8,38]*	*[8,38]*	*[8,40]*	*[8,40]*	*[8,40]*

[1] In the GEDA study, persons ≥18 years are interviewed. It was assumed that the probabilities of adolescents aged 15 to 17 are similar to the probabilities analyzed for the age group 18 to 29 years.
[2] For cases of unknown age, the probabilities of all study participants of the GEDA study were evaluated.

2) General parameters

General assumptions regarding the sensitivity of laboratory analyses for the detection of *Campylobacter* spp. and the proportion of CE cases with bloody diarrhea in the population were updated (Table J). The probabilities of analyzing a stool sample for *Campylobacter* spp. and of reporting a positive result were based on expert opinion as proposed by Haagsma et al. [35].

Table J. Distribution functions of general parameters for the reconstruction of the surveillance pyramid

Parameter		Distribution	Ref.
p_{st}	Probability of analyzing stool samples for *Campylobacter* spp.	Beta(9.9, 0.1)	[35]
p_{rep}	Probability of reporting a positive laboratory result	Beta(9.9, 0.1)	[35]
$sens$	Sensitivity of laboratory analysis for the detection of *Campylobacter* spp.	Uniform(0.723,0.899)[1]	[41,42]
p_{bd}	Proportion of *Campylobacter* enteritis cases with bloody diarrhea in the population	Beta(1.22,5.75)	see Table K

[1] Lowest and highest sensitivity of different methods tested: Sensitivity of stool culture was 72.3-89.9 % [41,42]; sensitivity of commercial stool antigen tests was 78.8-87.5 % [41].

Haagsma et al. [35] performed a literature review of outbreak studies to derive the proportion of CE cases with bloody diarrhea. They included published reports from 1965 to 2008 of 15 separate outbreaks described in 14 publications [43–56].

Based on this search, MEDLINE via PubMed was searched in April 2025 using the following terms:

("Disease Outbreaks"[MeSH Terms] OR outbreak[Title/Abstract]) AND ("Diarrhea"[MeSH Terms] OR diarr OR "gastroenteritis"[MeSH Terms] OR "Gastrointestinal Hemorrhage"[MeSH Terms] OR blood*) AND ("Campylobacter"[MeSH Terms] OR "Campylobacter Infections"[MeSH Terms] OR campylobacter*[Title/Abstract]).*

All results since 2008 (n = 172) were screened. 16 new reports on the proportion of bloody diarrhea in CE cases were identified [57–72]; among them, two publications were follow-up reports of previously described outbreaks and did not provide new data on the proportion of bloody diarrhea [57,61].

In addition, the search engine Google Scholar was used for a targeted search for outbreaks in Germany (*"Campylobacter Ausbruch Deutschland"*), which found one report [73].

Altogether, 30 published reports of the proportions of bloody diarrhea in CE cases were found (Table K). In accordance with Haagsma et al. [35], a Beta distribution was fitted to the proportions by maximum likelihood estimation in R 4.4.1 using the package fitdistrplus [74]. The resulting parameters were $\alpha = 1.216$ and $\beta = 5.749$ (Table J).

Table K. Outbreak studies reporting the proportion of bloody diarrhea in *Campylobacter* enteritis cases

Author and year	Country, year of outbreak	Cases with diarrhea *[n]*	Cases with bloody diarrhea	
			[n]	*[%]*
Allerberger et al. 2003 [43]	Austria, Germany, Liechtenstein, 2001	5	1	20.0
CDC 1983 [44]	USA, 1983	16	3	18.8
CDC 1983 [44]	USA, 1983	18	3	16.7
CDC 1986 [45]	USA, 1985	22	5	22.7
CDC 1998 [46]	USA, 1996	14	3	21.4
CDC 2002 [47]	USA, 2001	70	17	24.3
KDHE 2008; CDC 2009 [48,57]	USA, 2007	66	18	27.3
Engberg et al. 1998 [49]	Denmark, 1995-1996	65	22	33.8
Fahey et al. 1995 [50]	UK, 1992	110	14	12.7
Kornblatt et al. 1985 [51]	USA, 1981	33	4	12.1
Kuusi et al. 2004 [52]	Finland, 2000	101	3	3.0
Mazick et al. 2006 [53]	Denmark, 2005	75	3	4.0
Olsen et al. 2001 [54]	USA, 1998	129	14	10.9
Richardson et al. 2007 [55]	UK, 2000	281	22	7.8
Roels et al. 1998 [56]	USA, 1995	45	10	22.2
Campagnolo et al. 2018 [58]	USA, 2016	3	1	33.3
Caron et al. 2023 [59]	USA, 2021	8	4	50.0
Edwards et al. 2014 [60]	UK, 2011	45	8	17.8
Emberland et al. 2022; Hyllestad et al. 2020 [61,62]	Norway, 2019	1,626	113	6.9
Gardner et al. 2011 [63]	USA, 2008	98	20	20.4
Griffiths et al. 2010 [64]	UK, 2008	151	15	9.9
Inns et al. 2010 [65]	UK, 2010	24	5	20.8
Jakopanec et al. 2008 [66]	Norway, 2007	95	2	2.1
McAllister et al. 2023 [67]	Australia, 2022	26	6	23.1
Moffatt et al. 2010 [68]	Australia, 2005	25	2	8.0
Stuart et al. 2010 [69]	Canada, 2007	225	29	12.9
Wardak et al. 2008 [70]	Poland, 2006	4	0	0.01[1]
Wikswo et al. 2022 [71]	USA, 2009-2019	5,362	1,437	26.8
Zeigler et al. 2014 [72]	USA, 2012	18	6	33.3
Thurm et al. 1999 [73]	Germany, 1997	186	30	16.1

[1] 0.01 % added for the fitting of the Beta distribution.

3) Calculation of underestimated *Campylobacter* enteritis cases

The underestimated mild, moderate and severe CE cases per age group were calculated as follows [35]:

1) Formula for the estimation of underreported moderate cases per age group:

$$n_{mod\ UR} = \frac{n_{rep} - n_{hosp}}{(p_{bd} * st_{bd} + (1 - p_{bd}) * st_{nbd}) * p_{st} * p_{rep} * sens} - (n_{rep} - n_{hosp})$$

2) Formula for the estimation of underreported severe cases per age group:

$$n_{sev\ UR} = \frac{n_{hosp}}{st_{hosp} * p_{st} * p_{rep} * sens} - n_{hosp}$$

3) Formula for the estimation of under-ascertained mild cases per age group:

$$n_{mild\ UA} = \frac{n_{rep} + n_{mod\ UR} + n_{sev\ UR}}{p_{bd} * GP_{bd} + (1 - p_{bd}) * GP_{nbd}} - n_{rep} - n_{mod\ UR} - n_{sev\ UR}$$

where

- n_{rep} is the age- and gender-specific number of notified cases (Table B),
- n_{hosp} is the age- and gender-specific number of reported hospitalized cases (Table B),
- p_{st} is the probability of analyzing stool samples for *Campylobacter* spp. (Table J),
- p_{rep} is the probability of reporting a positive laboratory result (Table J),
- $sens$ is the sensitivity of laboratory analysis for the detection of *Campylobacter* spp. (Table J),
- p_{bd} is the proportion of *Campylobacter* enteritis cases with bloody diarrhea in the population (Table J),
- GP_{bd} is the probability of visiting a GP with bloody diarrhea (Table I),
- GP_{nbd} is the probability of visiting a GP with non-bloody diarrhea (Table I),
- st_{bd} is the probability of submitting a stool sample at GP, with bloody diarrhea (Table I),
- st_{nbd} is the probability of submitting a stool sample at GP, with non-bloody diarrhea (Table I), and
- st_{hosp} is the probability of submitting a stool sample in hospitalized patients (Table I).

References

1. Statistisches Bundesamt (Destatis). Bevölkerung: Deutschland, Stichtag, Altersjahre, Nationalität/Geschlecht/Familienstand. 2024 [cited 15 Aug 2024]. Available from: https://www-genesis.destatis.de/datenbank/online/statistic/12411/table/12411-0006.
2. Statistisches Bundesamt (Destatis). Sterbetafeln 2020/2022. Ergebnisse aus der laufenden Berechnung von Periodensterbetafeln für Deutschland und die Bundesländer. 2023 [cited 15 Aug 2023]. Available from: https://www.destatis.de/DE/Themen/Gesellschaft-Umwelt/Bevoelkerung/Sterbefaelle-Lebenserwartung/Publikationen/Downloads-Sterbefaelle/statistischer-bericht-sterbetafeln-5126207227005.xlsx.
3. Robert Koch-Institut (RKI). SurvStat@RKI 2.0 [cited 5 Jun 2025]. Available from: https://survstat.rki.de.
4. Statistisches Bundesamt (Destatis). Gesundheit. Tiefgegliederte Diagnosedaten der Krankenhauspatientinnen und -patienten. 2024 [cited 15 Aug 2024]. Available from: https://www.statistischebibliothek.de/mir/receive/DESerie_mods_00000950.
5. Statistisches Bundesamt (Destatis). Statistischer Bericht. Diagnosen der Krankenhauspatienten 2022. 2024 [cited 15 Aug 2024]. Available from: https://www.destatis.de/DE/Themen/Gesellschaft-Umwelt/Gesundheit/Krankenhaeuser/Publikationen/Downloads-Krankenhaeuser/statistischer-bericht-diagnosedaten-5231301227015.xlsx.
6. Statistisches Bundesamt (Destatis). Gesundheit. Ergebnisse der Todesursachenstatistik für Deutschland. 2024 [cited 15 Aug 2024]. Available from: https://www.statistischebibliothek.de/mir/receive/DESerie_mods_00000958.
7. Gesundheitsberichterstattung des Bundes. Sterbefälle, Sterbeziffern (je 100.000 Einwohner, altersstandardisiert). 2024 [cited 15 Aug 2024]. Available from: https://www.gbe-bund.de.
8. Robert Koch-Institute (RKI). German Health Update 2009 (GEDA 2009). Department of Epidemiology and Health Monitoring. Available from: https://doi.org/10.7797/26-200809-1-1-2.
9. Rosner BM, Schielke A, Didelot X, Kops F, Breidenbach J, Willrich N, et al. A combined case-control and molecular source attribution study of human Campylobacter infections in Germany, 2011-2014. Sci Rep. 2017; 7:5139. doi: 10.1038/s41598-017-05227-x.
10. Lackner J, Weiss M, Müller-Graf C, Greiner M. The disease burden associated with Campylobacter spp. in Germany, 2014. PLoS One. 2019; 14:e0216867. doi: 10.1371/journal.pone.0216867.
11. European Centre for Disease Prevention and Control (ECDC). ECDC BCoDE toolkit [software application]. Version 2.0.0. 2020 [cited 9 Nov 2020]. Available from: https://www.ecdc.europa.eu/en/publications-data/toolkit-application-calculate-dalys.
12. Schorling E, Knorr S, Lick S, Steinberg P, Brüggemann DA. Probability of sequelae following Campylobacter spp. infections: Update of systematic reviews and meta-analyses. Public Health Challenges. 2023; 2:e145. doi: 10.1002/puh2.145.
13. Hense S, Schink T, Kreisel SH, Marcelon L, Simondon F, Tahden M, et al. Estimation of background incidence rates of Guillain-Barré syndrome in Germany - a retrospective cohort study with electronic healthcare data. Neuroepidemiology. 2014; 43:244–52. doi: 10.1159/000369344.
14. Havelaar AH, Wit MAS de, van Koningsveld R. Health burden in the Netherlands (1990-1995) due to infections with thermophilic Campylobacter species. National Institute of Public Health and the Environment (RIVM); 2000. Available from: https://www.rivm.nl/bibliotheek/rapporten/284550004.pdf.
15. Mangen M, Havelaar AH, Wit GA de. Campylobacteriosis and sequelae in the Netherlands. Estimating the disease burden and the cost-of illness. National Institute for Public Health and the Environment (RIVM); 2004. Available from: https://www.rivm.nl/bibliotheek/rapporten/250911004.pdf.
16. Korinthenberg R, Mönting JS. Natural history and treatment effects in Guillain-Barré syndrome: a multicentre study. Archives of Disease in Childhood. 1996; 74:281–7. doi: 10.1136/adc.74.4.281.
17. Schessl J, Luther B, Kirschner J, Mauff G, Korinthenberg R. Infections and vaccinations preceding childhood Guillain-Barré syndrome: a prospective study. Eur J Pediatr. 2006; 165:605–12. doi: 10.1007/s00431-006-0140-1.
18. Doets AY, Verboon C, van den Berg B, Harbo T, Cornblath DR, Willison HJ, et al. Regional variation of Guillain-Barré syndrome. Brain. 2018; 141:2866–77. doi: 10.1093/brain/awy232.
19. Sturm A, Atreya R, Bettenworth D, Bokemeyer B, Dignaß A, Ehehalt R, et al. Aktualisierte S3-Leitlinie "Diagnostik und Therapie des Morbus Crohn". Deutsche Gesellschaft für Gastroenterologie, Verdauungs- und Stoffwechselkrankheiten (DGVS), editor. 2021. Available from: https://www.awmf.org/uploads/tx_szleitlinien/021-004l_S3_Morbus_Crohn_Diagnostik_Therapie_2021-08.pdf.
20. Kucharzik T, Dignass AU, Atreya R, Bokemeyer B, Esters P, Herrlinger K, et al. Aktualisierte S3-Leitlinie Colitis ulcerosa – Living Guideline. Z Gastroenterol. 2020; 58:e241-e326. doi: 10.1055/a-1296-3444.

21. Barbara G, Grover M, Bercik P, Corsetti M, Ghoshal UC, Ohman L, et al. Rome Foundation Working Team Report on post-infection irritable bowel syndrome. Gastroenterology. 2019; 156:46-58.e7. doi: 10.1053/j.gastro.2018.07.011.
22. Marshall JK, Thabane M, Garg AX, Clark WF, Moayyedi P, Collins SM. Eight year prognosis of postinfectious irritable bowel syndrome following waterborne bacterial dysentery. Gut. 2010; 59:605–11. doi: 10.1136/gut.2009.202234.
23. Neal KR, Barker L, Spiller RC. Prognosis in post-infective irritable bowel syndrome: a six year follow up study. Gut. 2002; 51:410–3. doi: 10.1136/gut.51.3.410.
24. Youn YH, Kim HC, Lim HC, Park JJ, Kim J-H, Park H. Long-term Clinical Course of Post-infectious Irritable Bowel Syndrome After Shigellosis: A 10-year Follow-up Study. J Neurogastroenterol Motil. 2016; 22:490–6. doi: 10.5056/jnm15157.
25. Haagsma JA, Maertens de Noordhout C, Polinder S, Vos T, Havelaar AH, Cassini A, et al. Assessing disability weights based on the responses of 30,660 people from four European countries. Popul Health Metr. 2015; 13:10. doi: 10.1186/s12963-015-0042-4.
26. Schorling E, Lick S, Steinberg P, Brüggemann DA. Health care utilizations and costs of Campylobacter enteritis in Germany: A claims data analysis. PLoS One. 2023; 18:e0283865. doi: 10.1371/journal.pone.0283865.
27. Statistisches Bundesamt (Destatis). Harmonisierter Verbraucherpreisindex: Deutschland, Jahre. 2025 [cited 5 Jun 2025]. Available from: https://www-genesis.destatis.de/datenbank/online/statistic/61121/table/61121-0001/.
28. Statistisches Bundesamt (Destatis). Arbeitskosten je geleistete Stunde (Jahresschätzung): Deutschland, Jahre, Wirtschaftsbereiche. 2025 [cited 5 Jun 2025]. Available from: https://www-genesis.destatis.de/datenbank/online/table/62431-0001/.
29. Eurostat. Average number of usual weekly hours of work in main job [LFSA_EWHUIS]. 2025 [cited 5 Jun 2025]. Available from: https://ec.europa.eu/eurostat/databrowser/view/LFSA_EWHUIS/default/table.
30. Statistisches Bundesamt (Destatis). Erwerbstätigenquoten der 15- bis unter 65-Jährigen mit Kindern unter 18 Jahren. [cited 26 Apr 2022]. Available from: https://www-genesis.destatis.de/datenbank/online/statistic/12211/table/12211-9048/.
31. Wittig R, Albers L, Koletzko S, Saam J, Kries R von. Pediatric Chronic Inflammatory Bowel Disease in a German Statutory Health INSURANCE-Incidence Rates From 2009 to 2012. J Pediatr Gastroenterol Nutr. 2019; 68:244–50. doi: 10.1097/MPG.0000000000002162.
32. Velasco-Benítez CA, Collazos-Saa LI, García-Perdomo HA. A systematic review and meta-analysis in schoolchildren and adolescents with functional gastrointestinal disorders according to Rome IV criteria. Arq Gastroenterol. 2022; 59:304–13. doi: 10.1590/S0004-2803.202202000-53.
33. Koskull S von, Truckenbrodt H, Holle R, Hörmann A. Incidence and prevalence of juvenile arthritis in an urban population of southern Germany: a prospective study. Ann Rheum Dis. 2001; 60:940–5. doi: 10.1136/ard.60.10.940.
34. Institute for Health Metrics and Evaluation (IHME). Global Burden of Disease Study 2019 (GBD 2019) Data Input Sources Tool [cited 7 Dec 2022]. Available from: https://ghdx.healthdata.org/gbd-2019/data-input-sources.
35. Haagsma JA, Geenen PL, Ethelberg S, Fetsch A, Hansdotter F, Jansen A, et al. Community incidence of pathogen-specific gastroenteritis: reconstructing the surveillance pyramid for seven pathogens in seven European Union member states. Epidemiol Infect. 2013; 141:1625–39. doi: 10.1017/S0950268812002166.
36. Lange C, Jentsch F, Allen J, Hoebel J, Kratz AL, Lippe E von der, et al. Data Resource Profile: German Health Update (GEDA)--the health interview survey for adults in Germany. Int J Epidemiol. 2015; 44:442–50. doi: 10.1093/ije/dyv067.
37. Wilking H, Spitznagel H, Werber D, Lange C, Jansen A, Stark K. Acute gastrointestinal illness in adults in Germany: a population-based telephone survey. Epidemiol Infect. 2013; 141:2365–75. doi: 10.1017/S0950268813000046.
38. Scavia G, Baldinelli F, Busani L, Caprioli A. The burden of self-reported acute gastrointestinal illness in Italy: a retrospective survey, 2008-2009. Epidemiol Infect. 2012; 140:1193–206. doi: 10.1017/S0950268811002020.
39. Eurostat. Consultation of a medical doctor (in private practice or as outpatient) per inhabitant [hlth_hc_phys]. 2022 [cited 5 Dec 2023]. Available from: https://ec.europa.eu/eurostat/databrowser/view/hlth_hc_phys/default/table.
40. Kaiser P, Borte M, Zimmer K-P, Huppertz H-I. Complications in hospitalized children with acute gastroenteritis caused by rotavirus: a retrospective analysis. Eur J Pediatr. 2012; 171:337–45. doi: 10.1007/s00431-011-1536-0.

41. Fitzgerald C, Patrick M, Gonzalez A, Akin J, Polage CR, Wymore K, et al. Multicenter Evaluation of Clinical Diagnostic Methods for Detection and Isolation of Campylobacter spp. from Stool. J Clin Microbiol. 2016; 54:1209–15. doi: 10.1128/JCM.01925-15.
42. Buss JE, Cresse M, Doyle S, Buchan BW, Craft DW, Young S. Campylobacter culture fails to correctly detect Campylobacter in 30% of positive patient stool specimens compared to non-cultural methods. Eur J Clin Microbiol Infect Dis. 2019; 38:1087–93. doi: 10.1007/s10096-019-03499-x.
43. Allerberger F, Al-Jazrawi N, Kreidl P, Dierich MP, Feierl G, Hein I, et al. Barbecued chicken causing a multi-state outbreak of Campylobacter jejuni enteritis. Infection. 2003; 31:19–23. doi: 10.1007/s15010-002-3088-8.
44. Centers for Disease Control and Prevention (CDC). Campylobacteriosis Associated with Raw Milk Consumption -- Pennsylvania. MMWR Morb Mortal Wkly Rep. 1983; 32:337–8.
45. Centers for Disease Control and Prevention (CDC). Campylobacter outbreak associated with raw milk provided on a dairy tour--California. MMWR Morb Mortal Wkly Rep. 1986; 35:311–2.
46. Centers for Disease Control and Prevention (CDC). Outbreak of Campylobacter enteritis associated with cross-contamination of food--Oklahoma, 1996. MMWR Morb Mortal Wkly Rep. 1998; 47:129–31.
47. Centers for Disease Control and Prevention (CDC). Outbreak of Campylobacter jejuni infections associated with drinking unpasteurized milk procured through a cow-leasing program--Wisconsin, 2001. MMWR Morb Mortal Wkly Rep. 2002; 51:548–9.
48. Kansas Department of Health & Environment (KDHE). Outbreak of Campylobacter jejuni Infections Associated with Consumption of Cheese Made from Raw Milk – Western Kansas, 2007. 2008. Available from: https://www.kdhe.ks.gov/DocumentCenter/View/7425/Outbreak-of-Campylobacter-Jejuni-Infections-Associated-with-Consumption-of-Cheese-Made-From-Raw-Milk-in-Western-Kansas-PDF.
49. Engberg J, Gerner-Smidt P, Scheutz F, Møller Nielsen E, On SLW, Mølbak K. Water-borne Campylobacter jejuni infection in a Danish town---a 6-week continuous source outbreak. Clin Microbiol Infect. 1998; 4:648–56. doi: 10.1111/j.1469-0691.1998.tb00348.x.
50. Fahey T, Morgan D, Gunneburg C, Adak GK, Majid F, Kaczmarski E. An outbreak of Campylobacter jejuni enteritis associated with failed milk pasteurisation. J Infect. 1995; 31:137–43. doi: 10.1016/s0163-4453(95)92160-5.
51. Kornblatt AN, Barrett T, Morris GK, Tosh FE. Epidemiologic and laboratory investigation of an outbreak of Campylobacter enteritis associated with raw milk. Am J Epidemiol. 1985; 122:884–9. doi: 10.1093/oxfordjournals.aje.a114171.
52. Kuusi M, Klemets P, Miettinen I, Laaksonen I, Sarkkinen H, Hänninen ML, et al. An outbreak of gastroenteritis from a non-chlorinated community water supply. J Epidemiol Community Health. 2004; 58:273–7. doi: 10.1136/jech.2003.009928.
53. Mazick A, Ethelberg S, Nielsen EM, Mølbak K, Lisby M. An outbreak of Campylobacter jejuni associated with consumption of chicken, Copenhagen, 2005. Euro Surveill. 2006; 11:137–9.
54. Olsen SJ, Hansen GR, Bartlett L, Fitzgerald C, Sonder A, Manjrekar R, et al. An outbreak of Campylobacter jejuni infections associated with food handler contamination: the use of pulsed-field gel electrophoresis. J Infect Dis. 2001; 183:164–7. doi: 10.1086/317657.
55. Richardson G, Thomas DR, Smith RMM, Nehaul L, Ribeiro CD, Brown AG, et al. A community outbreak of Campylobacter jejuni infection from a chlorinated public water supply. Epidemiol Infect. 2007; 135:1151–8. doi: 10.1017/S0950268807007960.
56. Roels TH, Wickus B, Bostrom HH, Kazmierczak JJ, Nicholson MA, Kurzynski TA, et al. A foodborne outbreak of Campylobacter jejuni (O:33) infection associated with tuna salad: a rare strain in an unusual vehicle. Epidemiol Infect. 1998; 121:281–7. doi: 10.1017/s0950268898001174.
57. Centers for Disease Control and Prevention (CDC). Campylobacter jejuni infection associated with unpasteurized milk and cheese--Kansas, 2007. MMWR Morb Mortal Wkly Rep. 2009; 57:1377–9.
58. Campagnolo ER, Philipp LM, Long JM, Hanshaw NL. Pet-associated Campylobacteriosis: A persisting public health concern. Zoonoses Public Health. 2018; 65:304–11. doi: 10.1111/zph.12389.
59. Caron G, Viveiros B, Slaten C, Borkman D, Miller A, Huard RC. Campylobacter jejuni Outbreak Linked to Raw Oysters in Rhode Island, 2021. J Food Prot. 2023; 86:100174. doi: 10.1016/j.jfp.2023.100174.
60. Edwards DS, Milne LM, Morrow K, Sheridan P, Verlander NQ, Mulla R, et al. Campylobacteriosis outbreak associated with consumption of undercooked chicken liver pâté in the East of England, September 2011: identification of a dose-response risk. Epidemiol Infect. 2014; 142:352–7. doi: 10.1017/S0950268813001222.
61. Emberland KE, Wensaas K-A, Litleskare S, Iversen A, Hanevik K, Langeland N, et al. Clinical features of gastroenteritis during a large waterborne Campylobacter outbreak in Askøy, Norway. Infection. 2022; 50:343–54. doi: 10.1007/s15010-021-01652-3.

62. Hyllestad S, Iversen A, MacDonald E, Amato E, Borge BÅS, Bøe A, et al. Large waterborne Campylobacter outbreak: use of multiple approaches to investigate contamination of the drinking water supply system, Norway, June 2019. Euro Surveill. 2020; 25. doi: 10.2807/1560-7917.ES.2020.25.35.2000011.
63. Gardner TJ, Fitzgerald C, Xavier C, Klein R, Pruckler J, Stroika S, et al. Outbreak of campylobacteriosis associated with consumption of raw peas. Clin Infect Dis. 2011; 53:26–32. doi: 10.1093/cid/cir249.
64. Griffiths SL, Salmon RL, Mason BW, Elliott C, Thomas DR, Davies C. Using the internet for rapid investigation of an outbreak of diarrhoeal illness in mountain bikers. Epidemiol Infect. 2010; 138:1704–11. doi: 10.1017/S0950268810001561.
65. Inns T, Foster K, Gorton R. Cohort study of a campylobacteriosis outbreak associated with chicken liver parfait, United Kingdom, June 2010. Euro Surveill. 2010; 15. doi: 10.2807/ese.15.44.19704-en.
66. Jakopanec I, Borgen K, Vold L, Lund H, Forseth T, Hannula R, et al. A large waterborne outbreak of campylobacteriosis in Norway: the need to focus on distribution system safety. BMC Infect Dis. 2008; 8:128. doi: 10.1186/1471-2334-8-128.
67. McAllister J, Gregory J, Adamopoulos J, Walsh M, Stylianopoulos A, Arnold A-L, et al. A foodborne outbreak of campylobacteriosis at a wedding - Melbourne, Australia, 2022. Communicable diseases intelligence. 2023; 47. doi: 10.33321/cdi.2023.47.10.
68. Moffatt CRM, Cameron S, Mickan L, Givney RC. Campylobacter jejuni gastroenteritis at an Australian boarding school: consistency between epidemiology, flaA typing, and multilocus sequence typing. Foodborne Pathog Dis. 2010; 7:1285–90. doi: 10.1089/fpd.2009.0468.
69. Stuart TL, Sandhu J, Stirling R, Corder J, Ellis A, Misa P, et al. Campylobacteriosis outbreak associated with ingestion of mud during a mountain bike race. Epidemiol Infect. 2010; 138:1695–703. doi: 10.1017/S095026881000049X.
70. Wardak S, Szych J, Sadkowska-Todys M. The first report on Campylobacter coli family outbreak detected in Poland in 2006. Euro Surveill. 2008; 13.
71. Wikswo ME, Roberts V, Marsh Z, Manikonda K, Gleason B, Kambhampati A, et al. Enteric illness outbreaks reported through the National Outbreak Reporting System, United States, 2009-19. Clin Infect Dis. 2022; 74:1906–13. doi: 10.1093/cid/ciab771.
72. Zeigler M, Claar C, Rice D, Davis J, Frazier T, Turner A, et al. Outbreak of campylobacteriosis associated with a long-distance obstacle adventure race--Nevada, October 2012. MMWR Morb Mortal Wkly Rep. 2014; 63:375–8.
73. Thurm V, Dinger E, Lyytikäinen O, Petersen L, Wiebelitz A, Lange D, et al. Infektionsepidemiologie lebensmittelbedingter Campylobacter-Infektionen. Bundesgesundheitsblatt - Gesundheitsforschung - Gesundheitsschutz. 1999; 42:206–11. doi: 10.1007/s001030050085.
74. Delignette-Muller ML, Dutang C. fitdistrplus : An R Package for Fitting Distributions. J Stat Soft. 2015; 64:1–34. doi: 10.18637/jss.v064.i04.
