# Supplementary material for: The disease and economic burden of notified and underestimated Campylobacter enteritis cases and associated sequelae in Germany
Source: PLoS One. 2025 Sep 4;20(9):e0331298. doi: 10.1371/journal.pone.0331298 (PMC12410716; doi:10.1371/journal.pone.0331298)
Supplement: S2 Appendix — (PDF) [file pone.0331298.s002.pdf]

Additional results

for the disease and economic burden of

Campylobacter enteritis in Germany

by Schorling E, Lick S, Rosner B, Knorr S, Wilking H, Steinberg P, Brüggemann DA

Table A. Health and economic burden associated with *Campylobacter* enteritis cases

	notified			underestimated			total		
	mean	95% CI		mean	95% CI		mean	95% CI	
***Campylobacter* enteritis (CE)**									
**mild CE**									
Cases	-			264,561	263,319	265,803	264,561	263,319	265,803
DALY				174	170	178	174	170	178
YLD				174	170	178	174	170	178
YLL				0			0		
Total costs [in € 1000]				23,490	23,350	23,630	23,490	23,350	23,630
Direct costs				-					
Indirect costs				23,490	23,350	23,630	23,490	23,350	23,630
**moderate CE**									
Cases	42,464			135,107	134,584	135,629	177,571	177,048	178,093
DALY	109	109	109	353	352	355	462	460	464
YLD	109	109	109	353	352	355	462	460	464
YLL	0			0			0		
Total costs [in € 1000]	27,545	27,524	27,567	86,109	85,778	86,439	113,654	113,318	113,990
Direct costs	9,362	9,356	9,368	27,450	27,348	27,552	36,812	36,709	36,915
Indirect costs	18,183	18,166	18,201	58,658	58,426	58,891	76,842	76,604	77,080
**severe CE**									
Cases	11,114			13,155	13,043	13,266	24,269	24,157	24,380
fatal cases	*6*			*4*	*4*	*4*	*10*	*10*	*10*
DALY	90	90	90	85	84	86	175	174	176
YLD	47	47	47	57	57	58	104	103	104
YLL	43	43	43	28	28	28	71	71	71
Total costs [in € 1000]	37,302	37,295	37,309	41,905	41,566	42,244	79,207	78,868	79,547
Direct costs	30,202	30,197	30,207	33,919	33,645	34,194	64,121	63,847	64,396
Indirect costs	7,100	7,095	7,105	7,986	7,920	8,053	15,086	15,019	15,153

Mean and 95% confidence intervals (CI) of 10,000 simulations. Extrapolation is based on the mean of notified *Campylobacter* enteritis (CE) cases between 2018 and 2022.
DALY: disability-adjusted life years; YLD: years lived with disability; YLL: years of life lost.

Table B. Health and economic burden associated with sequelae of *Campylobacter* enteritis

	following notified CE			following underestimated CE			total			sensitivity analysis[1]		
	mean	95% CI		mean	95% CI		mean	95% CI		mean	95% CI	
**Reactive arthritis**												
Cases	927	910	944	2,577	2,528	2,627	3,505	3,439	3,571	8,110	7,955	8,266
DALY	191	185	197	534	516	552	725	701	749	1,682	1,624	1,739
YLD	191	185	197	534	516	552	725	701	749	1,682	1,624	1,739
YLL	0			0			0			0		
Total costs [in € 1000]	587	566	608	1,670	1,607	1,733	2,257	2,173	2,340	5,251	5,054	5,449
Direct costs	482	465	500	1,347	1,295	1,399	1,829	1,760	1,898	4,240	4,078	4,402
Indirect costs	105	100	109	323	310	336	428	410	445	1,012	970	1,054
**Guillain-Barré syndrome**												
Cases	35	35	36	277	272	282	312	306	318	see total		
DALY	39	38	40	362	355	369	401	393	408			
YLD	32	32	33	280	275	285	312	306	319			
YLL	6	6	7	82	80	83	88	86	90			
Total costs [in € 1000]	1,769	1,727	1,811	15,425	15,104	15,746	17,194	16,833	17,555			
Direct costs	1,000	977	1,023	8,638	8,460	8,816	9,638	9,438	9,838			
Indirect costs	769	748	790	6,787	6,624	6,950	7,556	7,372	7,740			
**mild GBS**												
Cases	6	6	6	47	46	48	53	52	54	see total		
DALY	0	0	0	1	1	1	1	1	1			
YLD	0	0	0	1	1	1	1	1	1			
YLL	0			0			0					
Total costs [in € 1,000]	72	69	74	580	561	599	652	630	673			
Direct costs	38	37	40	307	297	317	345	334	356			
Indirect costs	33	32	35	274	264	284	307	296	318			
**severe GBS**												
Cases	30	29	30	230	225	234	259	255	264	see total		
fatal cases	*1*	*1*	*1*	*11*	*11*	*11*	*12*	*12*	*12*			
DALY	39	38	40	360	353	368	399	391	407			
YLD	32	32	33	279	273	284	311	305	317			
YLL	6	6	7	82	80	83	88	86	90			
Total costs [in € 1,000]	1,697	1,657	1,737	14,845	14,538	15,152	16,542	16,197	16,887			
Direct costs	961	939	983	8,332	8,161	8,503	9,293	9,101	9,485			
Indirect costs	736	715	756	6,513	6,358	6,669	7,249	7,074	7,424			

	following notified CE			following underestimated CE			total			sensitivity analysis[1]		
	mean	95% CI		mean	95% CI		mean	95% CI		mean	95% CI	
**Inflammatory bowel diseases**												
Cases	308	303	312	852	839	864	1,159	1,143	1,176	2,681	2,641	2,721
DALY	718	707	728	2,015	1,984	2,045	2,732	2,692	2,772	6,401	6,305	6,497
YLD	718	707	728	2,015	1,984	2,045	2,732	2,692	2,772	6,401	6,305	6,497
YLL	0			0			0			0		
Total costs [in € 1,000]	4,587	4,519	4,655	14,246	14,026	14,465	18,833	18,548	19,117	44,235	43,551	44,920
Direct costs	1,452	1,428	1,476	4,074	4,004	4,144	5,526	5,433	5,620	12,950	12,726	13,173
Indirect costs	3,135	3,088	3,181	10,172	10,015	10,329	13,307	13,105	13,508	31,286	30,800	31,771
**Crohn's disease**												
Cases	114	112	116	316	309	322	429	421	438	993	973	1,013
DALY	265	260	270	746	731	761	1,012	992	1,032	2,371	2,323	2,418
YLD	265	260	270	746	731	761	1,012	992	1,032	2,371	2,323	2,418
YLL	0			0			0			0		
Total costs [in € 1,000]	1,695	1,660	1,729	5,396	5,280	5,512	7,091	6,941	7,241	16,718	16,360	17,077
Direct costs	350	343	357	984	963	1,005	1,333	1,306	1,361	3,124	3,058	3,190
Indirect costs	1,345	1,317	1,373	4,412	4,315	4,510	5,757	5,633	5,882	13,595	13,295	13,895
**Ulcerative colitis**												
Cases	194	190	198	536	526	547	730	716	744	1,688	1,654	1,721
DALY	452	443	461	1,268	1,243	1,293	1,720	1,687	1,754	4,030	3,950	4,111
YLD	452	443	461	1,268	1,243	1,293	1,720	1,687	1,754	4,030	3,950	4,111
YLL	0			0			0			0		
Total costs [in € 1,000]	2,892	2,835	2,950	8,850	8,669	9,030	11,742	11,505	11,978	27,517	26,954	28,080
Direct costs	1,103	1,080	1,125	3,090	3,024	3,156	4,193	4,104	4,281	9,826	9,616	10,036
Indirect costs	1,790	1,754	1,826	5,760	5,641	5,878	7,549	7,396	7,703	17,691	17,325	18,057
**Irritable bowel syndrome**												
Cases	2,387	2,345	2,430	6,615	6,493	6,737	9,002	8,839	9,166	20,800	20,416	21,183
DALY	554	540	569	1,541	1,499	1,582	2,095	2,039	2,151	4,845	4,714	4,976
YLD	554	540	569	1,541	1,499	1,582	2,095	2,039	2,151	4,845	4,714	4,976
YLL	0			0			0			0		
Total costs [in € 1,000]	2,148	2,090	2,205	6,671	6,487	6,855	8,818	8,579	9,058	20,939	20,361	21,516
Direct costs	424	413	436	1,180	1,146	1,213	1,604	1,559	1,649	3,710	3,605	3,816
Indirect costs	1,723	1,677	1,769	5,491	5,338	5,644	7,214	7,017	7,412	17,229	16,750	17,708

Mean and 95% confidence intervals (CI) of 10,000 simulations. Extrapolation is based on the mean of notified *Campylobacter* enteritis (CE) cases between 2018 and 2022. DALY and costs in future years were discounted with 3 %.

[1] Sensitivity analysis: assuming that reactive arthritis, inflammatory bowel diseases and irritable bowel syndrome might also develop following mild CE cases.

DALY: disability-adjusted life years; YLD: years lived with disability; YLL: years of life lost.

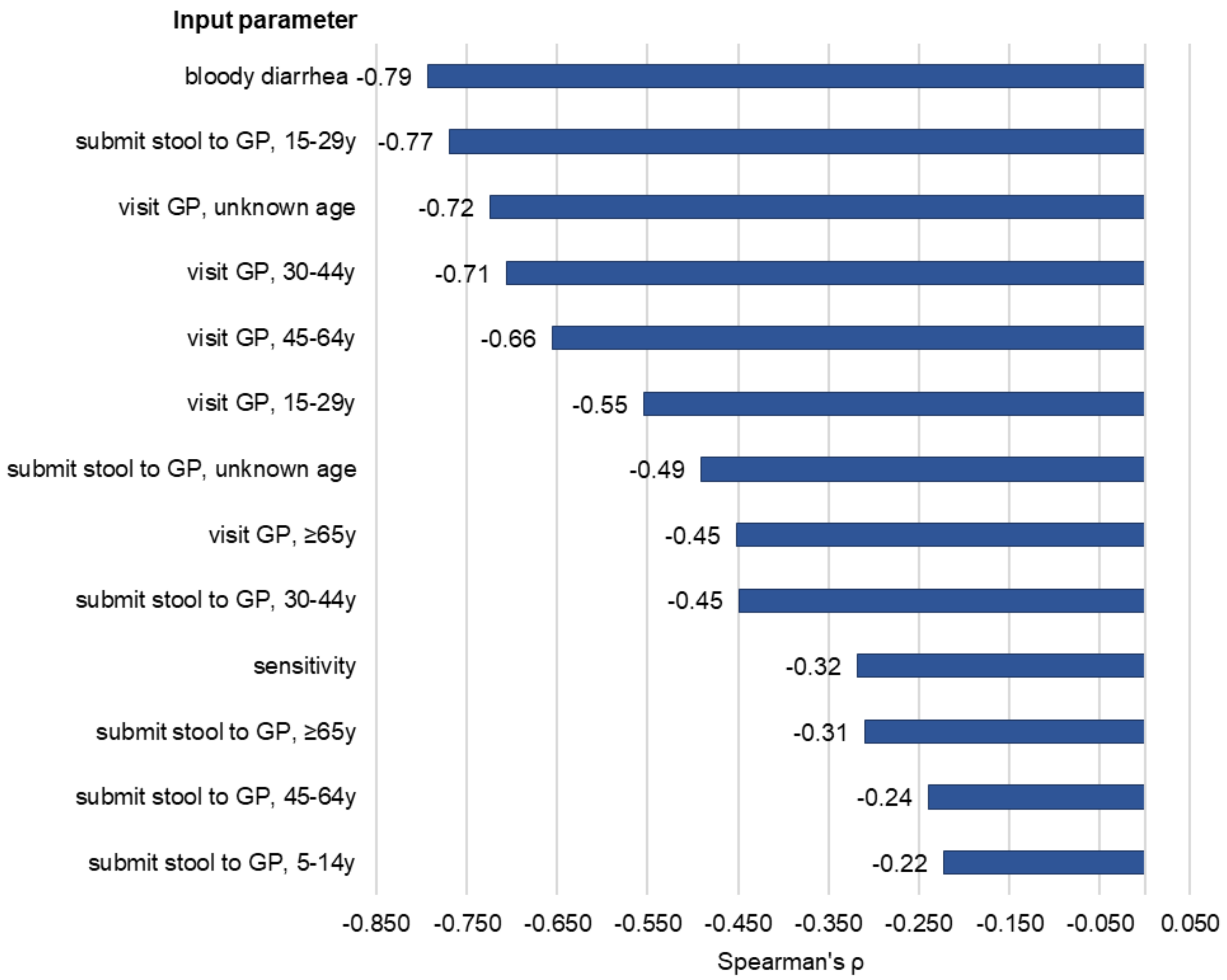

Fig A. Correlation between the different input parameters for the model and the resulting underestimated Campylobacter enteritis cases in Germany

Results of 10,000 iterations; Spearman's rank correlation. Input parameters with ρ ≥ |0.2| are displayed.
GP: general practitioner; y: years.

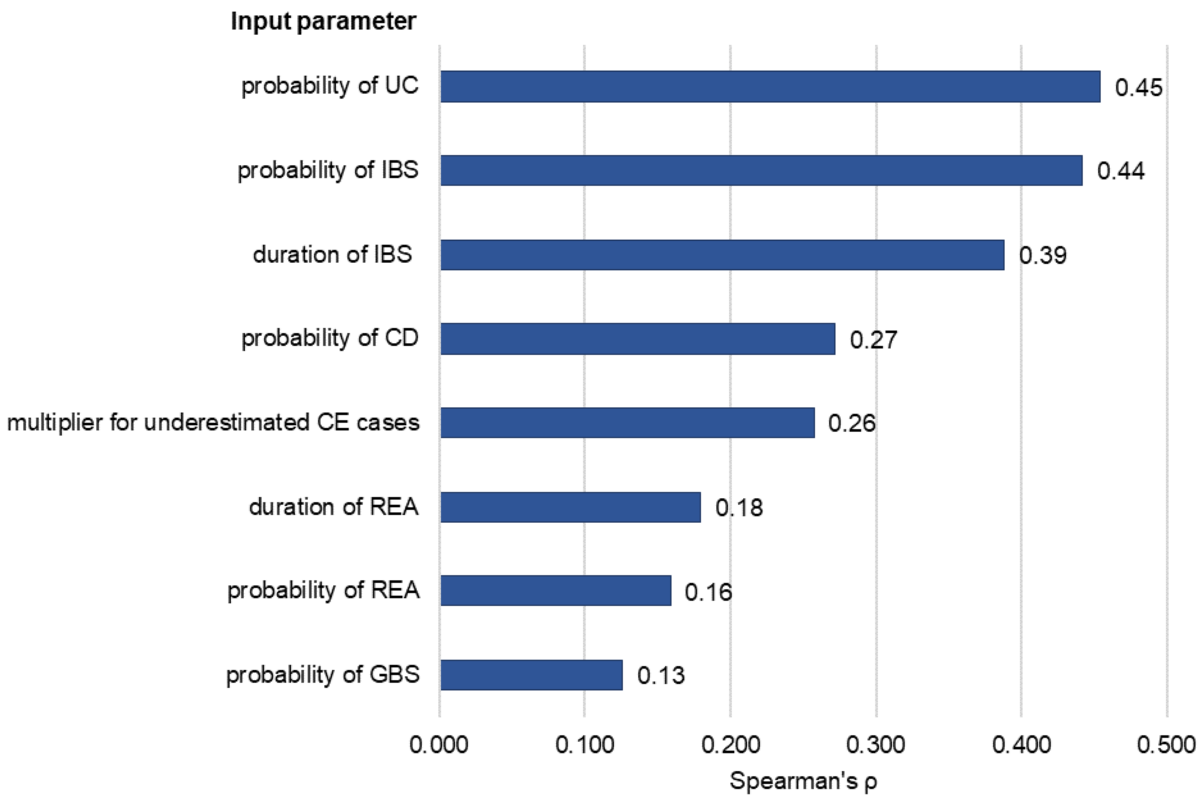

Fig B. Correlation between the different input parameters for the model and the resulting total DALY of *Campylobacter* enteritis and associated sequelae in Germany

Results of 10,000 iterations; Spearman's rank correlation between input parameters and total DALY (disability-adjusted life years; sum of years lived with disability and years of life lost), discounted with 3 %. Input parameters with $\rho \geq |0.1|$ are displayed.
CD: Crohn's disease; CE: *Campylobacter* enteritis; GBS: Guillain-Barré syndrome; IBS: irritable bowel syndrome; REA: reactive arthritis; UC: ulcerative colitis

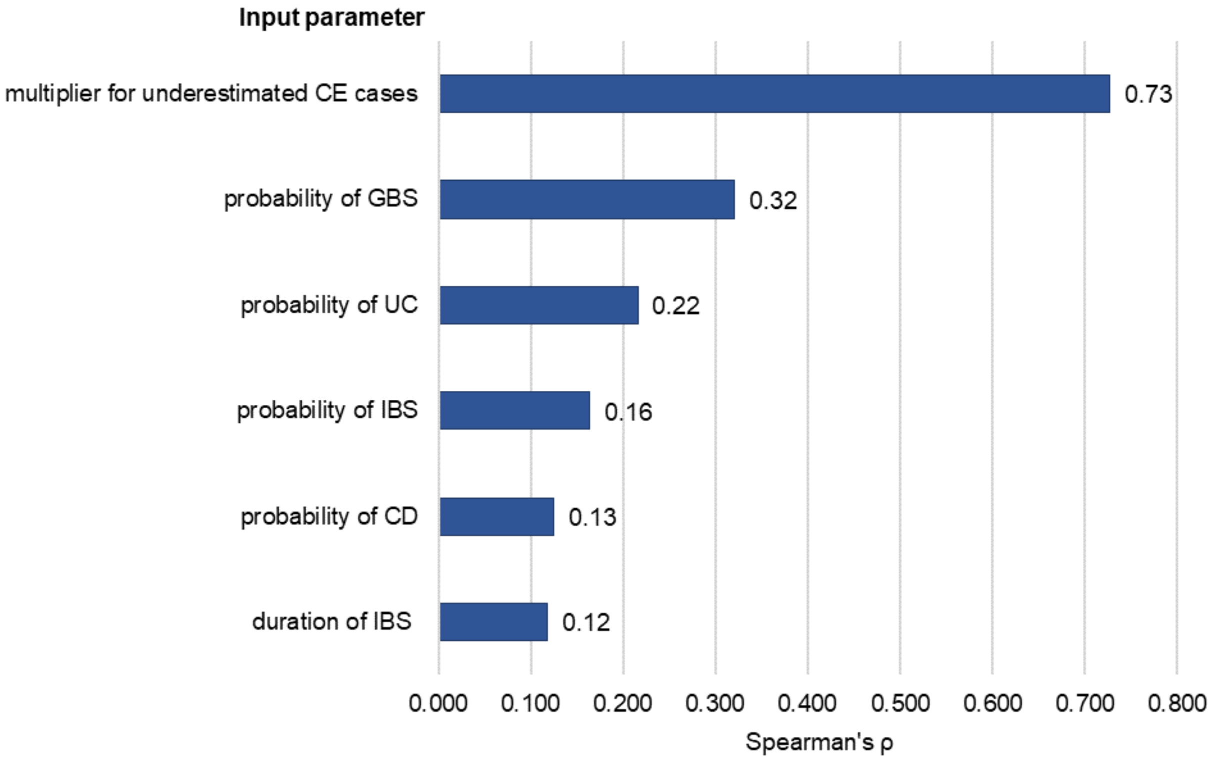

Fig C. Correlation between the different input parameters for the model and the resulting total costs of *Campylobacter* enteritis and associated sequelae in Germany

Results of 10,000 iterations; Spearman's rank correlation between input parameters and total costs (sum of direct and indirect costs), discounted with 3 %. Input parameters with $\rho \geq |0.1|$ are displayed.
CD: Crohn's disease; CE: *Campylobacter* enteritis; GBS: Guillain-Barré syndrome; IBS: irritable bowel syndrome
